# Convergent genomic diversity and novel BCAA metabolism in intrahepatic cholangiocarcinoma

**DOI:** 10.1038/s41416-023-02256-4

**Published:** 2023-04-19

**Authors:** Akihiro Kitagawa, Tsuyoshi Osawa, Miwa Noda, Yuta Kobayashi, Sho Aki, Yusuke Nakano, Tomoko Saito, Dai Shimizu, Hisateru Komatsu, Maki Sugaya, Junichi Takahashi, Keisuke Kosai, Seiichiro Takao, Yushi Motomura, Kuniaki Sato, Qingjiang Hu, Atsushi Fujii, Hiroaki Wakiyama, Taro Tobo, Hiroki Uchida, Keishi Sugimachi, Kohei Shibata, Tohru Utsunomiya, Shogo Kobayashi, Hideshi Ishii, Takanori Hasegawa, Takaaki Masuda, Yusuke Matsui, Atsushi Niida, Tomoyoshi Soga, Yutaka Suzuki, Satoru Miyano, Hiroyuki Aburatani, Yuichiro Doki, Hidetoshi Eguchi, Masaki Mori, Keiichi I. Nakayama, Teppei Shimamura, Tatsuhiro Shibata, Koshi Mimori

**Affiliations:** 1 Department of Surgery, Kyushu University Beppu Hospital, 4546 Tsurumihara, Beppu, 874-0838 Japan; 2 Department of Gastroenterological Surgery, Graduate School of Medicine, Osaka University, 2-2 Yamadaoka, Suita, 565-0871 Japan; 3 Division of Integrative Nutiriomics and Oncology, Research Center for Advanced Science and Technology, The University of Tokyo, Tokyo, 153-8904 Japan; 4 Department of Clinical Laboratory Medicine, Kyushu University Beppu Hospital, 4546 Tsurumihara, Beppu, 874-0838 Japan; 5 Department of Gastroenterological Surgery, Oitaken Koseiren Tsurumi Hospital, 4333 Tsurumihara, Beppu, 874-8585 Japan; 6 Department of Surgery, Oita Prefectural Hospital, 2-8-1 Bunyo, Oita, 870-8511 Japan; 7 Department of Frontier Science for Cancer and Chemotherapy, Graduate School of Medicine, Osaka University, 2-2 Yamadaoka, Suita, 565-0871 Japan; 8 Division of Health Medical Computational Science, Health Intelligence Center, Institute of Medical Science, The University of Tokyo, 4-6-1 Shirokanedai, Minato-ku, Tokyo, 108-8639 Japan; 9 Division of Systems Biology, Nagoya University Graduate School of Medicine, Nagoya, Aichi 466-8550 Japan; 10 Institute for Advanced Biosciences, Keio University, Kakuganji, Tsuruoka, 997-0052 Japan; 11 Laboratory of Systems Genomics, Department of Computational Biology and Medical Sciences, Graduate School of Frontier Sciences, The University of Tokyo, 5-1-5 Kashiwanoha, Kashiwa, Chiba, 277-8561 Japan; 12 Laboratory of DNA Information Analysis, Human Genome Center, Institute of Medical Science, The University of Tokyo, 4-6-1 Shirokanedai, Minato-ku, Tokyo, 108-8639 Japan; 13 Genome Science Division, Research Center for Advanced Science and Technology, The University of Tokyo, 4-6-1 Komaba, Meguro-ku, Tokyo, 153-8904 Japan; 14 Department of Surgery and Science, Graduate School of Medical Science, Kyushu University, 3-1-1 Maidashi, Higashi-ku, Fukuoka, Fukuoka, 812-8582 Japan; 15 Department of Molecular and Cellular Biology, Medical Institute of Bioregulation, Kyushu University, 3-1-1 Maidashi, Higashi-ku, Fukuoka, Fukuoka, 812-8582 Japan; 16 Division of Cancer Genomics, National Cancer Center Research Institute, 5-1-1 Tsukiji, Chuo-ku, Tokyo, 104-0045 Japan

**Keywords:** Cancer metabolism, Evolutionary genetics, Liver cancer

## Abstract

**Background:**

Driver alterations may represent novel candidates for driver gene-guided therapy; however, intrahepatic cholangiocarcinoma (ICC) with multiple genomic aberrations makes them intractable. Therefore, the pathogenesis and metabolic changes of ICC need to be understood to develop new treatment strategies. We aimed to unravel the evolution of ICC and identify ICC-specific metabolic characteristics to investigate the metabolic pathway associated with ICC development using multiregional sampling to encompass the intra- and inter-tumoral heterogeneity.

**Methods:**

We performed the genomic, transcriptomic, proteomic and metabolomic analysis of 39–77 ICC tumour samples and eleven normal samples. Further, we analysed their cell proliferation and viability.

**Results:**

We demonstrated that intra-tumoral heterogeneity of ICCs with distinct driver genes per case exhibited neutral evolution, regardless of their tumour stage. Upregulation of BCAT1 and BCAT2 indicated the involvement of ‘Val Leu Ile degradation pathway’. ICCs exhibit the accumulation of ubiquitous metabolites, such as branched-chain amino acids including valine, leucine, and isoleucine, to negatively affect cancer prognosis. We revealed that this metabolic pathway was almost ubiquitously altered in all cases with genomic diversity and might play important roles in tumour progression and overall survival.

**Conclusions:**

We propose a novel ICC onco-metabolic pathway that could enable the development of new therapeutic interventions.

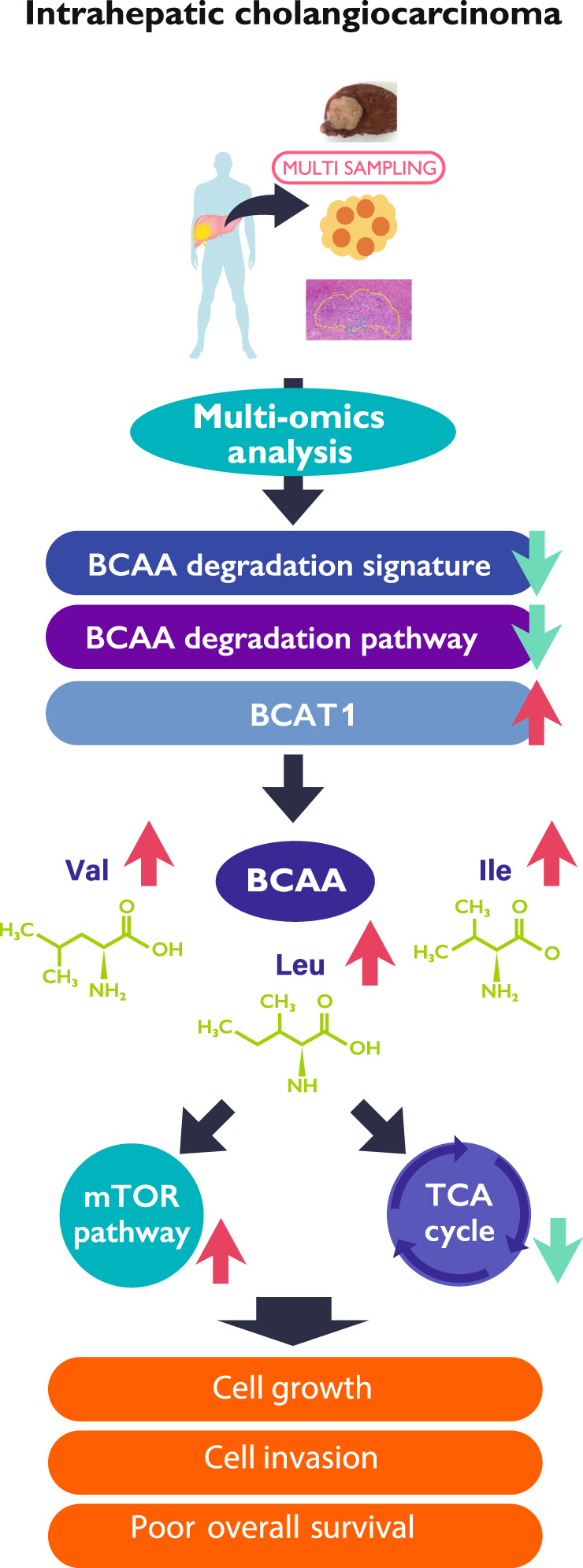

## Background

Intrahepatic cholangiocarcinoma (ICC), the second most frequent type of malignancy originating from the liver, accounts for ~15% of primary liver cancers [1]. Over the past few decades, the incidence of ICC has been steadily increasing [2]. Surgical resection remains the primary treatment of choice, but most patients with ICC are refractory to treatment and have a dismal outcome; the 5-year overall survival rates after resection vary at 30‒35% [3]. The commonly used treatment modalities, such as chemotherapy or radiotherapy, exhibit purely palliative effects on ICC, enabling only a limited improvement in survival. Therefore, the pathogenesis and onco-metabolic changes of ICC need to be understood to develop new treatment strategies.

Intra- and inter-tumoral heterogeneity are increasingly attracting attention in cancer research. During the development and progression of cancer, intra-tumoral heterogeneity (ITH) complicates the diagnosis and treatment of cancer. Inter-tumoral heterogeneity results in the range of tumour genotypes found in different patients. Various tumours with genomic diversity induce substantial downstream molecular heterogeneity that leads to different oncogenic metabolic pathways. In recent years, genomic analysis has provided insights into the genetic landscape of ICC [4]. The driver alterations identified in these studies may represent novel candidates for driver gene-guided therapy. There are numerous treatment strategies for personalised therapy; thus far, only a few have been able to demonstrate improvement in survival compared to conventional treatment with standard chemotherapy [5]. Fibroblast growth factor receptor 2 inhibitors were the first to transform the clinical management of ICC, displaying the required efficacy in fusion-positive cases; however, those cases account for only 10–20% of the total [6], similar to cases with mutations in isocitrate dehydrogenase (IDH) [7]. Previously reported classifications of driver genes, including IDH, KRAS and TP53 were based on single sampling [8]. However, these studies did not elucidate the importance of the vast inter-tumoral heterogeneity in individual cases of ICC.

Cancer cells utilise altered metabolic pathways to facilitate the uptake and incorporation of abundant nutrients efficiently into core cellular molecules, such as nucleotides, amino acids, and lipids, for uninterrupted proliferation, and for survival in specific metabolic environments. This seems to be a universal characteristic of highly malignant tumours [9], independent of their carcinogenetic origin [10]. Metabolic reprogramming constitutes a part of the altered metabolic changes observed for decades [11]. However, whether this reprogramming is a general aspect of proliferation or an unintended consequence of aberrant signalling remains poorly understood; whether reprogramming is functionally involved in oncogenesis remains to be determined. For example, autophagy plays a critical role in glutamine metabolism, which is required for tumour survival in pancreatic ductal adenocarcinoma [12]. Coactivator SRC-2-dependent metabolic reprogramming mediates prostate cancer cell survival and metastasis and is considered a potential therapeutic target [13]. Thus, it is important to identify novel oncogenic factors that reprogramme metabolic pathways that influence ICC progression or malignancy.

Herein, we sampled ten multiregional ICC cases and used a multi-omics approach, including genomics, transcriptomics, proteomics, and metabolomics, to understand intra- and inter-tumoral heterogeneity. We aimed to determine whether the evolutionary process is neutral and analyse the onco-metabolic changes in the potential regulatory networks that underlie metabolic reprogramming in ICC, which may help elucidate new targetable pathways beyond both heterogeneities.

## Methods

### Ethics statement

The study design was approved by the institutional review boards and ethics committees of the following hospitals (Kyushu University Institutional Review Board: Protocol Number P-594-00; Osaka University Hospital Institutional Review Board: Protocol Number P-586; Oita Prefectural Hospital Institutional Review Board: Protocol Number P-26-19; Oitaken Koseiren Tsurumi Hospital Institutional Review Board: Protocol Number P-26-3-1; and Fukuoka City Hospital Institutional Review Board: Protocol Number P-15-E04). The study was conducted according to the principles of the Declaration of Helsinki. We obtained written informed consent from the 12 patients. No animal experiments were performed in this study. We performed whole-exome sequencing (WES), whole-transcriptome sequencing, proteome analysis and metabolome analysis on ten, eleven, ten, and ten of the ICC samples, respectively (Table 1).

**Table 1:** Detailed information of 12 cases.

Case	Aetiology of infection	sex	Age	Max size (cm)	NAC	Tumour type	Differentiation	T	N	M	Stage (UICC 8th edition)	Vp, Vv, Va	Prognosis
FS1	None	M	79	1.8	None	Mass forming type	Moderately	T1a	N0	M0	Stage IA	None	Alive without recurrence
FS2	None	M	80	1.5	None	Mass forming type	Moderately	T1a	N0	M0	Stage IA	None	Death
KS1	HBV + , HCV +	M	67	12	GS 2kur	Mass forming type	Moderately to poorly	T1b	N0	M0	Stage IB	None	Alive without recurrence
KS2	None	F	79	3.5	None	Mass forming and periductal infiltrating type	Well to moderately	T3	N0	M0	Stage IIIA	Vp1	Death
KS3	None	M	67	6	None	Mass forming type	Well to moderately	T2	N1	M1	Stage IV	Vv2	Death
KS4	None	F	70	6	GEM 3kur	Mass forming type	Well to moderately	T2	N1	M0	Stage IIIB	None	Death
KT1	None	F	64	5.4	None	Mass forming type	Moderately	T1b	N0	M0	Stage IB	None	Alive without recurrence
OK1	None	M	72	7.6	None	Mass forming type	Well	T1b	N0	M0	Stage IB	None	Death
OK2	None	M	78	4.4	None	Mass forming type	Moderately	T2	N0	M0	Stage II	Vp1	Death
OS1	None	M	76	8.8	None	Mass forming type	Moderately	T2	N1	M0	Stage IIIB	Vp1	Death
OS2	None	F	59	4.1	GCS 3kur	Mass forming type	Moderately	T2	N0	M0	Stage II	Vp1	Alive without recurrence
OS3	None	M	72	3.7	None	Mass forming type	Well to poorly	T1a	N1	M0	Stage IIIB	none	Death

Inclusion and exclusion criteria did not include special conditions, except unintended reasons for sampling, remaining sample volume, study design issues, etc.

### iMPAQT assay

We obtained 39 tumour samples and ten normal samples of ICC from ten cases for proteome analysis (Supplementary Table S1). Herein, we employed the in vitro proteome-assisted multiple reaction monitoring for protein absolute quantification (iMPAQT) [14] assay to perform global analysis for absolute quantification of protein expression simultaneously. The analysis was performed as described previously [14]. Briefly, frozen tissue was crushed using a bead shocker, lysed in 100 μL lysis buffer (containing 2% sodium dodecyl sulphate, 7 M urea, 100 mM Tris-HCl (pH 8.8); per 10 mg of tissue powder), and diluted with an equal amount of water. The protein concentrations in the samples were determined using the Bicinchoninic Acid assay (Bio-Rad Laboratories, Hercules, CA, USA). To block the cysteine/cysteine residues, we treated portions of the samples (200 μg of protein) with 5.0 mM Tris (2-carboxyethyl) phosphine hydrochloride (Thermo Fisher Scientific, Waltham, MA, USA) for 30 min at 37 °C and then performed alkylation with 10 mM 2-iodoacetoamide (Sigma-Aldrich, St. Louis, MO, USA) for 30 min at 20 °C. Next, these samples were subjected to acetone precipitation. The resulting pellet was resuspended in 100 μL digestion buffer (0.5 M triethylammonium bicarbonate). Each sample was digested with lysyl-endopeptidase (2 μg, Wako) for 3 h at 37 °C. Then, the samples were further digested with trypsin (4 μg, Thermo Fisher Scientific) for 14 h at 37 °C. The resulting cell digests were freeze-dried and labelled with the mTRAQ Δ0 (light) reagent. Each sample was spiked with synthetic peptides (Funakoshi and GenScript) for internal standard, which was with treated reductive alkylation and mTRAQ Δ4 (heavy) labelling. The samples were subjected to reversed-phase liquid chromatography followed by multiple reaction monitoring analysis. Experiments using mass spectrometry and pre-treatment were performed by Kyusyu Pro Search LLP (Fukuoka, Japan).

### Metabolome analysis

Metabolites present in cells or homogenised tumour tissue samples were quantified using capillary electrophoresis-mass spectrometry (Agilent Technologies, Santa Clara, CA, USA) as previously described [15]. Briefly, to analyse the cationic compounds, a fused silica capillary (50 µm i.d. × 100 cm) was used with 1 M formic acid as the electrolyte. Methanol/water (50% v/v) containing 0.1 µM hexakis (2,2-difluoroethoxy) phosphazene was delivered as the sheath liquid at 10 µL/min. Electrospray ionisation–time-of-flight mass spectrometry was performed in a positive ion mode, and the capillary voltage was set at 4 kV. Automatic recalibration of each acquired spectrum was achieved using the masses of the reference standards, (13 C isotopic ion of a protonated methanol dimer [2 MeOH+H] + , m/z 66.0632) and (hexakis[{2,2- difluoroethoxy} phosphazene +H] + , m/z 622.0290). To identify the metabolites, relative migration times of all peaks were calculated by normalisation to a reference compound, 3-aminopyrrolidine. The metabolites were identified by comparing their m/z values and relative migration times with the metabolite standards. Quantification was performed by comparing the peak areas with the calibration curves generated using internal standardisation techniques using methionine sulfone. The other conditions were identical to those previously described [15]. To analyse the anionic metabolites, a commercially available COSMO( + ) (chemically coated with cationic polymer) capillary (50 µm i.d. × 105 cm) (Nacalai Tesque, Kyoto, Japan) was used with a 50 mM ammonium acetate solution (pH 8.5) as the electrolyte. Methanol/5 mM ammonium acetate (50% v/v) containing 0.1 µM hexakis (2,2-difluoroethoxy) phosphazene was delivered as the sheath liquid at 10 µL/min. Electrospray ionisation–time-of-flight mass spectrometry was performed in a negative ion mode, and the capillary voltage was set at 3.5 kV. For anion analysis, trimesate and CAS were used as the reference and internal standards, respectively. The other conditions were identical to those described previously [16]. Raw capillary electrophoresis–time-of-flight mass spectrometry data were analysed using proprietary software Master Hands (ver, 2.17.0.10). Briefly, data processing for each experiment included data conversion, binning data into 0.02 m/z slices, baseline elimination, peak picking, integration, and elimination of redundant features to yield the possible peaks lists. Data matrices were generated using an alignment process based on corrected migration times, and metabolite names were assigned to the aligned peaks by matching the m/z and corrected migration times of our standards library. Relative peak areas were calculated based on the sample peak area-internal standard peak area ratio, and the metabolite concentrations were calculated based on the relative peak area between the sample and standard mixture.

### Cell proliferation assay

Cells were seeded on 96-well plates in a control medium at a concentration of 10^3^ cells/well. Following the attachment of the cells, the control medium was replaced with media with/without branched-chain amino acids (BCAA). The viability of the attached cells was measured at 24, 48 and 72 h following each treatment using the sulforhodamine B (SRB, Sigma-Aldrich) cell proliferation assay as previously described [17].

## Results

### Multi-omics analyses of the ICC samples

Following multiregional sampling of primary ICC cases, we performed multi-omics analyses, including genome, transcriptome, proteome, and metabolome analysis. We used 10 (67 samples), 11 (88 samples), 10 (49 samples) and 10 (49 samples) ICC cases for WES, whole-transcriptome sequencing, proteomic analysis, and metabolomic analysis, respectively (Table 1, Supplementary Table 1 and Supplementary Fig. S1).

### Multi-region sequencing of ten ICC cases

**Fig. 1:**
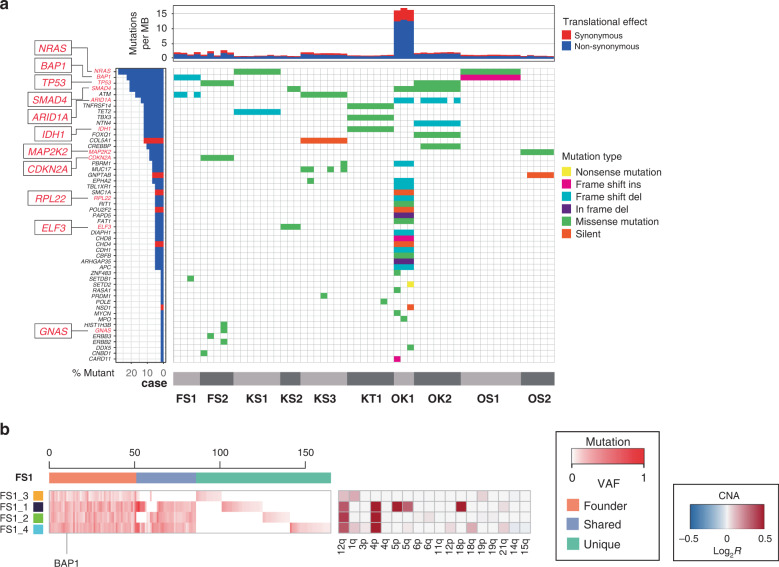
An integrated view of ITH in the cases in our study. **a** Mutational catalogue of 51 driver genes along with mutated allele frequency. Genes are sorted based on percentages of mutations per sample. **b** Multi-region mutation profiles of ICCs. Representative FS1 case was subjected to multi-region WES, and variant allele frequencies of all mutations, including short insertions/deletions, are presented as a heatmap for each case. Coloured bars on the top indicate three categories of mutations, i.e., ubiquitous, shared, and unique. Coloured bars on the left represent sample labels; colour similarity represents the similarity between mutation profiles. Previously reported driver genes with possible functional mutations, including non-synonymous SNV, stop–gain SNV, splicing SNV, or insertions/deletions, have been specified under each heatmap.

To characterise ITH in ICC, we performed multi-region WES of ten ICC cases. For each case, we sequenced three to nine multi-region tumour samples and a paired normal liver sample as a control, which amounted to 57 tumour samples and ten normal samples in total. WES, with a median fold coverage of 128.39 (range: 80.25–189.91), detected a median of 63 (range: 42–781) mutations for each sample (Fig. 1a, b and Supplementary Fig. S2A). The case OK1, harboured a median of 763 (range: 751–781) mutations; this was a hypermutated ICC case. Based on multi-region mutation profiles (Fig. 1a), mutations were categorised as either ubiquitous or heterogeneous. Herein, heterogeneous mutations were further subcategorised into shared mutations, which existed in some of the samples, and unique mutations, which were observed in a single sample. Polymerase chain reaction-based deep sequencing of randomly sampled mutations validated 100%, 100% and 94.2% ubiquitous, shared, and unique mutations, respectively. As expected, ICC exhibited more inter-tumoral heterogeneity than ITH, as ICC samples tended to harbour more heterogeneous mutations between cases than in a single case. As previously reported, KRAS, IDH, and TP53 mutations are mutually exclusive as such mutations may be complementary in a minority of cases, given their opposing nature—as oncogenes or tumour suppressors—depending on their temporal expression during cellular transformation [8]. No significant differences were observed in the spectra between ubiquitous and heterogeneous mutations across ten ICCs (Supplementary Fig. S2B).

Next, we estimated the copy-number alterations (CNAs) from WES data and analysed the multi-region CNA profiles. Similar to the mutations, CNAs correlated with ITH in ICC (Supplementary Fig. S2C). By focusing on the CNAs in chromosomal arms, we compared the distribution of the ubiquitous and heterogeneous CNAs between tumour stages. Overall, the numbers of heterogeneous CNAs in ICC were not significantly different from those observed during tumour progression (FS1 is a representative case; Fig. 1b and Supplementary Fig. S2D).

### Evolution of ten ICC cases

**Fig. 2:**
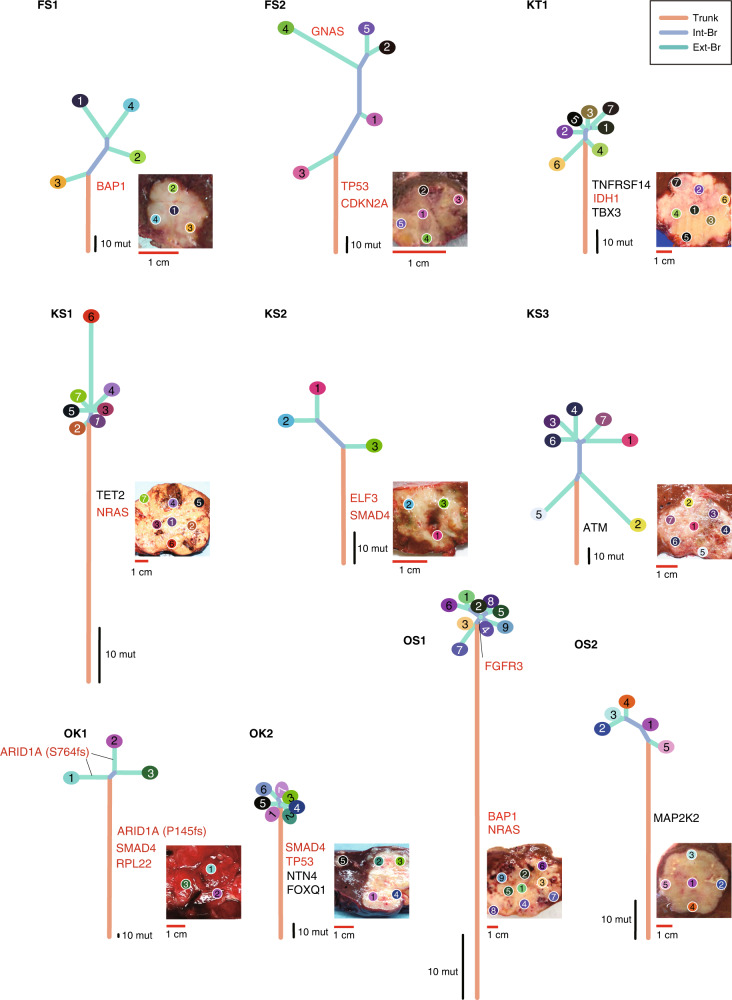
Evolutionary trees of ICCs. Ten evolutionary trees were constructed using the multi-region WES data with the Treeomics algorithm. Trunks, internal branches (int-Br) and external branches (ext-Br) generally correspond to ubiquitous, shared, and private mutations, respectively, whereas leaves correspond to the samples. The colours of the leaves are the same as the sample labels in Fig. 1 and Supplementary Fig. S2D. The lengths of the trunk and branches represent the number of mutations, and scales for ten mutations are shown near the roots of the evolutionary trees. Driver genes with possible functional mutations are mapped along the evolutionary trees. The image of each tumour is provided along with positions from which each sample was obtained. Red scale bars for one centimetre attempted with each photo.

Ten cases with ICC had a number of non-silent mutations in the known biliary tract cancer driver genes [18], such as SMAD4 (mutated in three cases), BAP1 (two cases), NRAS (two cases), TP53 (two cases), ELF3 (one case), CDKN2A (one case), FGFR3 (one case), GNAS (one case), IDH1 (one case), PIK3CA (one case) and RPL22 (one case) (Fig. 1b). The mutation rates of BAP1, KRAS, IDH1, TP53 and SMAD4 were consistent with those of previous reports on typical ICC [8]. We constructed the evolutionary trees for the ten ICCs using the Treeomics algorithm [19] to our multi-region sequencing data (Fig. 2). All ICC trees had ‘palm tree-like’ shapes (Neutral evolution), which were composed of long trunks and short branches; such trees are typically observed in advanced colorectal cancer [20]. Importantly, none of the ICC trees had ‘forked tree-like’ shapes (Darwinian evolution), which are usually observed in precancerous lesions of colorectal cancer and composed of short trunks and long branches [21]. To investigate the evolutionary history of each tumour, we mapped the known driver genes using possible functional mutations along with the evolutionary trees, which contained non-synonymous single-nucleotide variants (SNVs), stop–gain SNVs, splicing SNVs or insertions/deletions. These driver genes comprised those related to cancer [22], especially biliary tract cancer [18]. For example, the hypermutated OK1 case had two major branches, which had many mutations, as observed in the trunk. The first ARID1A mutation (P145 fs) was observed in the trunk, whereas the second ARID1A mutation (S764fs) was observed on the left (OK1_1) and right branches (OK1_2). We also found that both ARID1A (S764fs) mutations on the left and right branches had variant allele frequencies of ~0.4, whereas the first ARID1A mutation (P145fs) in the trunk had an allele frequency of ~0.50. The functionally non-synonymous hotspot mutations in IDH1 and IDH2 promote cholangiocarcinogenesis by suppressing hepatocyte differentiation [23]; therefore, the KT1 case had a ubiquitous IDH mutation in the trunk but not in the branch. These observations suggest that the two major sub-clones were subjected to different processes leading to biallelic inactivation of ARID1A; an additional mutation in the second allele was acquired in the left branch, whereas the loss of heterozygosity accompanying the first mutation occurred in the trunk. Thus, ICCs with distinct driver genes in the trunk (ex. FS1 case with BAP1, or OK2 case with SMAD4 and TP53)—by case—have neutral evolutionary phylogenetic trees, regardless of their tumour stage, and clustering cancer evolutionary trees [24] confirmed the independence of these patterns in the phylogenetic tree and clinicopathological factors (Supplementary Fig. S2E and Table 1).

### Significant metabolic changes in multi-sampling ICC tissues

**Fig. 3:**
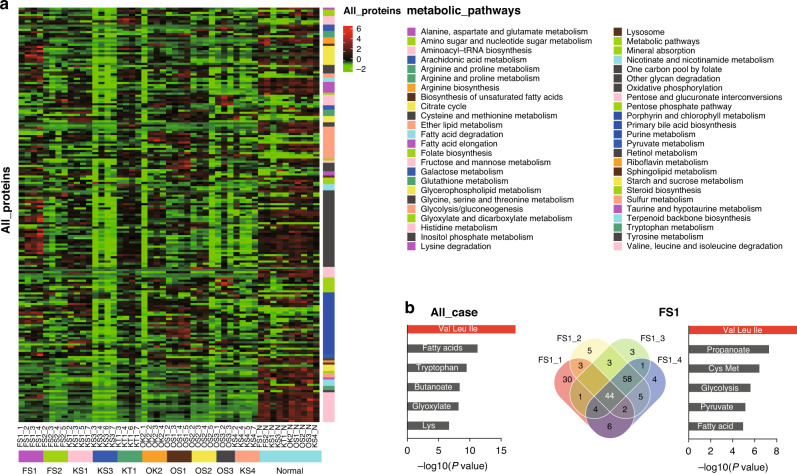
Proteome analysis of 195 primary metabolic proteins in our cohort. **a** Summary of the proteomes for all samples with four tumours and respective normal tissue samples. Clustering of the tissue samples below and metabolic pathways to the right. Red and green indicate increases and decreases, respectively. **b** Significant metabolic changes in multi-sampling ICC tissues (all case and representative FS1 case). The number indicates differentially expressed genes in common between two, three, or all four tumour sample tissues and a normal tissue sample. The barplot on the right side shows the significantly ranked metabolic pathways using KEGG pathway analysis for these genes (in common between all tumour samples and the normal tissue sample). Length of the bar indicates −log10 (*P* value).

As all ten cases had similar neutral evolutionary phylogenetic trees, regardless of distinct driver genes in the trunk (Fig. 2), we assumed that both heterogeneities might converge into ICC-specific onco-metabolic pathways. We have measured 324 primary metabolic proteins; among these, about 40 proteins containing more than 20% N/D (not detected) were excluded, therefore, we focused on 195 proteins (Supplementary Data 1). First, we analysed 39 ICC samples and ten normal samples from ten cases (four tumour samples and a respective normal sample per case, except with three tumour samples in OS3 case for sampling matter) during proteomic analysis using the iMPAQT method [14] (Fig. 3a). Absolute fold change >2 was used as the criteria to identify common differentially expressed genes between the four tumour samples and a normal tissue in each case. Then, we focused on the common metabolic proteins in each case as heterogenous proteins were observed in the samples of each case. Analysis of differential gene expression of matching tumour samples and normal liver tissue samples by iMPaLA (http://impala.molgen.mpg.de/) identified 29–66 genes annotated using the Kyoto Encyclopedia of Genes and Genomes (KEGG) for each case (FS1 is a representative case; Fig. 3b and Supplementary Fig. S3A). For example, in the FS1 case, we detected 44 common metabolic proteins, and ‘Val Leu Ile’ (BCAA degradation pathway) was the significantly affected (changed) metabolic pathway. Importantly, KEGG pathway analysis of these gene sets identified BCAA degradation as the most significant pathway in almost ten ICC cases, except the KT1 case (Supplementary Fig. S3A). Proteomic analysis revealed that 13 proteins involved in BCAA catabolism were widely downregulated in tumours relative to that in normal liver tissue (Fig. 3a and Supplementary Fig. S3B). Hence, genomically diverse ICCs tended to manifest ubiquitous changes in BCAA catabolism as depicted in the lowermost pathway in Fig. 3 and Supplementary Fig. S3.

### Altered gene expression in the BCAA degradation pathway

**Fig. 4:**
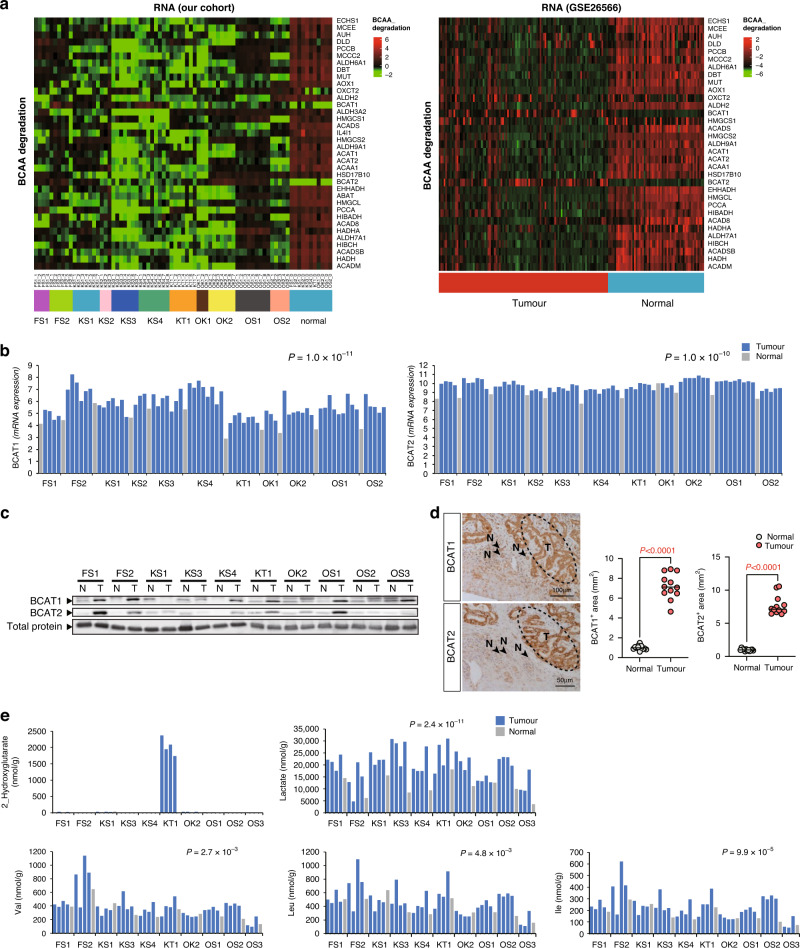
Transcriptomic, proteomic and metabolic analysis for BCAA catabolism and TCA cycle in our cohort and GSE26566. **a** Summary of the BCAA degradation pathway with 38 genes in our cohort and GSE26566. Clustering of the tissue samples below and genes to the right. Red and green indicate increases and decreases, respectively. **b** BCAT1 mRNA levels in 3‒9 tumour samples and a normal sample per case. The blue and grey bars represent tumour and normal samples, respectively. **c** Western blotting for the evaluation of BCAT1 and BCAT2 expression in the normal and tumour samples of all ten cases. **d** Immunohistochemical detection of BCAT1 and BCAT2 in the representative FS1 case of ICC. Original magnification: ×200. T tumour tissue, N normal liver tissue. Western blotting of the ICC samples. Quantification of immunohistochemical detection. **e** Metabolites include 2_Hydroxyglutarate, lactate, valine, leucine and isoleucine in 3‒4 tumour samples and one normal sample per case. The blue and grey bars represent tumour and normal samples, respectively. TCA, tricarboxylic acid.

Proteome analysis revealed the downregulation of the BCAA degradation pathways in tumours (Fig. 3 and Supplementary Fig. S3). Next, we investigated the changes in the transcriptome for intermediates of the BCAA degradation pathway to unravel the regulatory network underling ICC progression. Approximately 40 enzymes are involved in BCAA catabolism, and with the exception of the reversible transamination step performed using BCAT1 and BCAT2, 36 of these transcripts were found to be downregulated in tumours using transcriptome sequencing relative to transcripts in the paired normal tissues (Fig. 4a). Furthermore, these proteins were not heterogeneous, but were ubiquitous in our multi-sampling cohort. To focus on the large gene set as well as validate its findings and ensure robustness, we compared the changes in the expression of transcripts involved in BCAA catabolism in our cohort with those in an independent, well-characterised ICC cohort from the Gene Expression Omnibus (GSE26566; Fig. 4a). As expected, the expression of genes involved in the degradation of BCAA decreased in the tumour tissues in the GSE26566 cohort. However, BCAT1 and BCAT2 were upregulated in tumours in our cohort and GSE26566 (Fig. 4a).

Since BCAT1 and BCAT2 could not be quantified in iMPAQT method, western blotting were performed. BCAT1 and BCAT2 were tend to be highly expressed following western blotting and immunostaining, with the results being similar to those of the mRNA analysis (Fig. 4b–d and Supplementary Fig. S4A, B). By quantifying the expression of BCAT1/2 in each specimen, we confirmed that the expression of BCAT1 and BCAT2 was significantly increased in tumours (Fig. 4d and Supplementary Fig. S4C). Furthermore, immunostaining showed that both BCAT1 and BCAT2 were highly expressed in cancer cells when comparing noncancerous cholangiocytes (FS1 is a representative case; Fig. 4d and Supplementary Fig. S4A, B). In addition, the expression of the BCAA degradation pathway in extrahepatic cholangiocarcinoma and gallbladder cancer was similar to the expression in ICC, and in hepatocellular carcinoma (HCC), both BCAT1 and BCAT2 were highly expressed in tumour samples as previously reported [25] (Supplementary Fig. S5). We performed prognostic analyses of the BCAT1 and BCTA2 mRNA levels in the cohorts of Shibata, TCGA, and this study and found that the overall survival of the groups with high BCAT1 and BCAT2 expression (only in Shibata cohort) was significantly poorer than that of the groups with low BCAT1 and BCAT2 expression (Supplementary Fig. S6).

### Regulation of BCAA degradation pathway

The expression of genes involved in the degradation of BCAA decreased but BCAT1 and BCAT2 were upregulated in the tumour tissues. First, we analysed CNA in the gene expression of enzymes involved in the BCAA degradation pathway and found that some of BCAA catabolic enzymes showed partial somatic CNA loss in our cohort (Supplementary Fig. S7). In addition, we analysed the expression profiles of MYC downstream target genes (hereafter referred to as the MYC module activity) using the extraction of expression module algorithm. Our extraction of expression module analysis of MYC module activity could not explain this mechanism (Supplementary Fig. S8). Whereas no correlation was observed between the expression of BCAT1 and Musashi-2 (MSI2), BCAT2 expression was significantly positively correlated with that of MSI2 (Supplementary Fig. S9).

### Accumulation of valine, leucine and isoleucine

To explore the possibility of reprogramming BCAA metabolism, we performed capillary electrophoresis–time-of-flight mass spectrometry-based metabolome profiling of 3–4 tumour samples and one corresponding normal tissue per case (obtained from ten cases with ICC) (Supplementary Data 2). Contrary to most normal cells, many transformed cells derived a substantial amount of their energy from aerobic glycolysis, by converting most of the incoming glucose to lactate rather than metabolising it in the mitochondria through oxidative phosphorylation [26]. First, this metabolic analysis confirmed that the lactates had accumulated in all tumour samples, but not in normal samples (Paired *t* test, Fig. 4e). Furthermore, there was a remarkable accumulation of only 2-hydroxyglutarate in IDH-mutant tumours, similar to the KT1 case (Fig. 4e). A previous study demonstrated that IDH1 mutations result in the production of the onco-metabolite 2-hydroxyglutarate, and indicated that the excess 2-hydroxyglutarate contributes to the formation and malignant progression of ICC [27], gliomas [28], and chronic myelogenous leukaemia [29]. These results ensure the system’s reliability in mass accuracy. Among the changes in tumour samples, we observed a significant increase in BCAA (valine, leucine, and isoleucine) (Paired *t* test, Fig. 4e). In addition, BCAA degradation produces succinyl-CoA in the cells by generating propionyl-CoA from valine and isoleucine [30], subsequently providing substrates for the tricarboxylic acid cycle. However, significant depletion of the tricarboxylic acid cycle intermediate metabolites, including succinate, fumarate, and malate, was observed in the tumour samples (Paired *t* test, Supplementary Fig. S10). In addition, we measured blood BCAA levels in four healthy individuals and four ICC patients (FS2, KS4, OS2, OS3). We found that blood BCAA levels in ICC patients were not differed compared to healthy individuals (Supplementary Fig. S11).

### BCAA-stimulated cell growth and invasion via mTOR signalling in ICC

**Fig. 5:**
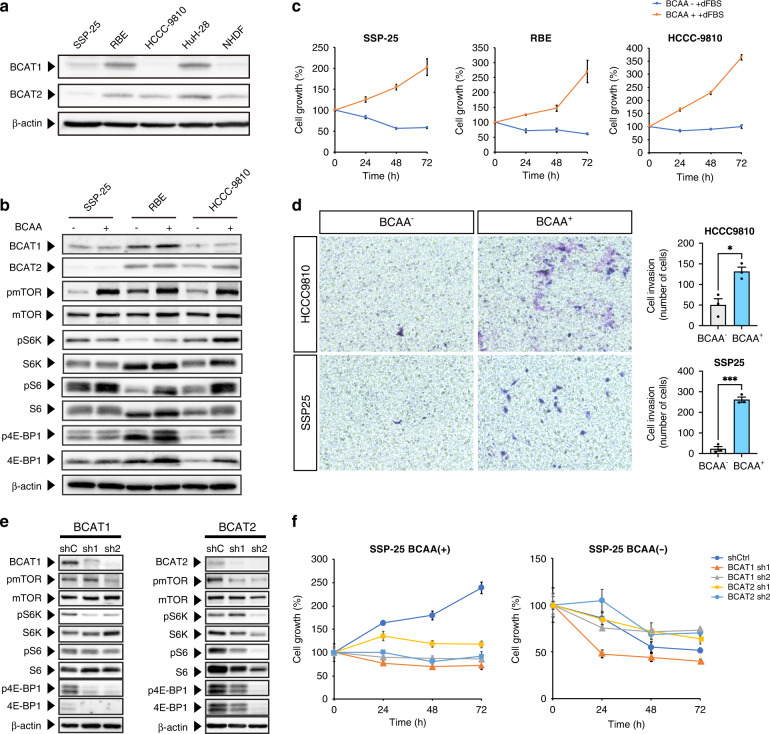
Branched-chain amino acids (BCAAs) increased cell growth and invasion via mTOR signalling in ICC cells. **a** Western blotting of BCAT1 and BCAT2 in ICC cells. BCAT1, BCAT2, and b-actin protein levels in human ICC cell lines (SSP-25, RBE and HCCC-9810) and normal human dermal fibroblast cells were evaluated using western blotting. **b** Western blotting of mTOR signalling proteins in ICC (SSP-25 and HCCC-9810) cells. **c** Cell growth of ICC cells with/without BCAAs. Cell growth of SSP-25 and HCCC-9810 cells was measured using SRB assay. **d** Cell invasion of ICC cells with/without BCAAs. Cell invasion was measured using SSP-25 and HCCC-9810 cells. **e** Western blotting of mTOR signalling proteins in BCAT1 and BCAT2 knockdown ICC (SSP-25) cells. **f** Cell growth of BCAT1 and BCAT2 knockdown ICC (SSP-25) cells. Cell growth of shBCAT1/shBCAT2-expressing SSP-25 cells was measured using SRB assay.

To examine whether BCAAs stimulate cell growth and invasion via mammalian target of rapamycin (mTOR) signalling in ICC, we examined the expression of the key catalytic enzymes of BCAAs, namely BCAT1 and BCAT2, in human ICC cell lines (SSP-25, RBE, and HCCC-9810) in comparison with their expression in normal human dermal fibroblast cells. We found that the expression of BCAT1 and BCAT2 increased in the ICC cells compared to primary normal dermal fibroblast (NHDF) cells (Fig. 5a). Further, to test whether BCAAs upregulate mTOR signalling in ICC cells, we performed western blot analysis. Phosphorylation of mTOR as well as that of the proteins downstream of mTOR in the mTOR pathway, including S6K, S6, and 4EBP-1, increased in the presence of BCAAs in SSP-25 and HCCC-9810 cells (Fig. 5b), concomitant with the observed increase in cell growth (Fig. 5c and Supplementary Fig. S12A), and invasion (Fig. 5d).

To test whether BCAT1/2 influence mTOR signalling in the presence of BCAAs in ICC cells, we knocked down either BCAT1 or BCAT2 in these cells. Knockdown of BCAT1 and BCAT2 led to a downregulation of mTOR signalling (Fig. 5e and Supplementary Fig. S12B) and a decrease in cell growth (Fig. 5f and Supplementary Fig. S12C), suggesting a role of BCAT1 and BCAT2 in the BCAA-mediated stimulation of mTOR signalling in ICC cells.

### Activated mTOR pathway in ICC

**Fig. 6:**
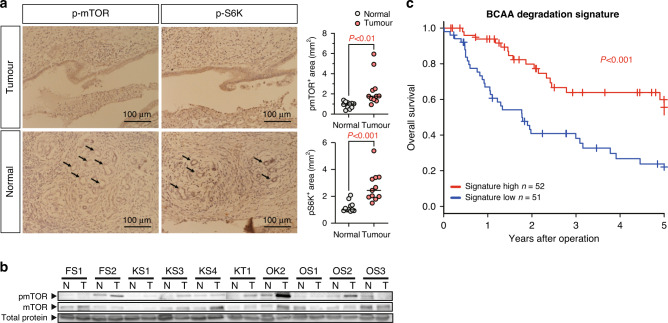
Overall impact of BCAA metabolism. **a** Immunohistochemical detection of phospho-mTOR and phospho-S6K expression in the representative FS2 case of ICC. Original magnifications: ×200 and ×400, respectively. T tumour tissue, N normal liver tissue. Quantification of the results of the immunohistochemical analyses. **b** Western blotting for the evaluation of p-mTOR and mTOR expression in the in normal and tumour samples of all ten cases. **c** Kaplan–Meier survival estimate curve for 103 cases ranked by the BCAA degradation signature.

Previous studies have revealed that chronic myeloid leukaemia and hepatocellular carcinoma display enhanced production of BCAAs, which promote mTOR activation [29]. Other reports have demonstrated that the activation of mTORC1 potently enhances cell growth and tumorigenesis in numerous human cancers and animal tumour models [31]. To evaluate the activation of the mTOR pathway, immunohistochemical analyses of p-mTOR and that of the mTORC1 downstream effector, p-S6k, were performed. We found that p-mTOR and p-S6k were highly expressed in tumour samples (FS2 is a representative case; Fig. 6a and Supplementary Fig. S13A, B). Given that the samples used for western blot analysis were obtained from bulk tissues, we often failed to detect a significant upregulation of p-mTOR and p-S6K expression. For IHC analysis, we quantified the expression of p-mTOR and p-S6K using individual specimen, and accordingly confirmed increases in the levels of p-mTOR and p-S6K expression in tumours (Fig. 6b and Supplementary Fig. S13C). These results were consistent with those of previous reports. Moreover, gene set enrichment analysis demonstrated that BCAT1, not BCAT2 expression in ICC was positively associated with the gene set HALLMARK_PI3K_AKT_MTOR_SIGNALING (NES: 1.52, *P* value: 0.0027) (Supplementary Fig. S14).

### Activity of BCAA degradation pathway correlates with case survival in ICC

To investigate the association of BCAA degradation pathway activity with prognosis in ICC cases, we analysed the matched transcriptome data and cohort survival information from 103 ICC tumours generated for previous studies [18], including 137 ICCs, 74 extrahepatic cholangiocarcinomas, and 28 gallbladder cancers (Shibata cohort). In the analysis, we selected 44 genes related to ‘BCAA degradation’ in the KEGG pathway database, and the activity of the BCAA degradation pathway was calculated for each case (Supplementary Table S2). Cases were then classified as ‘low BCAA degradation pathway activity’ for scores <50th percentile of the score and ‘high BCAA degradation pathway activity’ for scores >50th percentile of the score. When groups with high and low BCAA degradation pathway activity were compared, groups with low BCAA degradation pathway activity had lower overall survival rates (Fig. 6c). Furthermore, BCAA degradation signature in Shibata cohort was inversely correlated with MALAT1 (Supplementary Fig. S15).

## Discussion

We performed multi-region sampling through the systematic integration of the genome, transcriptome, proteome and metabolome of the resected tumours by sequencing different blocks from surgical specimens in ten ICC cases. By focusing on cancer metabolism, we identified the ICC-specific onco-metabolic pathway, BCAA catabolism, with vast genomic diversity among cases. We revealed that this metabolic pathway was almost ubiquitously altered in all ten cases and might play important roles in tumour progression and overall survival.

First, in WES analysis, ten ICCs had almost the same proportion of driver mutations as those observed in previous studies [8, 18], and the mutations were mutually exclusive, as expected. Notably, we found that ICCs have a slight degree of ITH in the driver genes compared to inter-tumour heterogeneity (Fig. 1a). Recent studies adopting multiregional WES have unveiled the complex ITH and evolutionary pattern in several types of cancers [32]. To identify processes involved in ICC progression, we observed the genomic history and evaluated the evolutionary pattern of the tumours. We demonstrated that ITH of ICCs with distinct driver genes per case exhibited neutral evolution, regardless of their tumour stage. Clustering cancer evolutionary trees [24] confirmed the independence of patterns on the phylogenetic tree and clinicopathological factors (Supplementary Fig. S2E and Table 1).

Although we demonstrated the intra- and inter-tumour heterogeneity in multiple genome dimensions, tumour development and progression in ICC have not been understood in detail. The correlations of these dimensions were not analysed because well-developed workflows are necessary for such extensive analyses ranging from the genome to the metabolome. Then, we performed multi-sampling of four samples per case to analyse the proteomic and metabolic profiles, considering that ICCs may have heterogenous metabolic proteins. KEGG pathway analysis of the results of our proteomic analysis revealed that the BCAA degradation pathway is the most significantly altered pathway, except in the KT1 case. The IDH-mutant type, observed in the KT1 case, was the only IDH mutant in ICC which seemed relatively rare in ICC, which did not alter the BCAA degradation pathway, and which produced specific metabolites that contributed to tumorigenesis [33]. We found that ~40 enzymes involved in BCAA catabolism were suppressed in tumours, with the exception of BCAT1 and BCAT2 that were upregulated in the transcriptome but not detected in the proteome. Satoh et al. revealed that the proto-oncogene protein MYC regulates global metabolic reprogramming in colorectal cancer by modulating 215 metabolic reactions [34]; however, our extraction of expression module analysis of MYC module activity could not explain this mechanism (Supplementary Fig. S8). We noted that some of the BCAA catabolic enzymes showed partly somatic copy-number variation loss in our cohort (Supplementary Fig. S7), as reported previously in the case of HCC [25]. BCAA degradation may be regulated by MALAT1 because the BCAA degradation signature in Shibata cohort was inversely correlated with MALAT1, which is known to regulate the phosphorylation of the SR protein, a splicing factor, to control alternative splicing (*R* = -0.669, *P* value  = 1.03 × 10^−14^, Supplementary Fig. S15) [35].

Metabolic pathway reprogramming has recently been reported to be a hallmark of cancer cell growth and survival; it supports the anabolic and energetic demands of these rapidly dividing cells [11, 13, 36]. Although BCAT1 catalyzes transamination in both directions and the breakdown of BCAAs is the predominant reaction in most cell types, BCAT1 generates BCAAs via the reverse reaction in cancer metabolic reprogramming [29]. Upregulation and functional requirements of BCAT1 have been reported for glioblastoma [37], colorectal tumours, and myeloid leukaemia [29]. MSI2, an oncogenic RNA-binding protein, is associated with the BCAT1 transcript and positively regulates its protein levels in those cancers [29]. Those reports demonstrate that the metabolic role of BCAT1 and BCAA seem distinct and dependent on cancer types. The expression of BCAT1 and BCAT2 was upregulated in tumours in the three cohorts. Notably, the expression of BCAT1 and BCAT2 was ubiquitously upregulated in tumour samples relative to that in the respective normal tissues in our multi-sampling cohort (Fig. 4b). No correlation was observed between the expression of BCAT1 and MSI2; however, BCAT2 expression was significantly positively correlated with that of MSI2 (Supplementary Fig. S9). Further, BCAT1 was negatively correlated with several miRNAs, which may be responsible for the high BCAT1 expression observed in TCGA cohort (Supplementary Data 3). We observed that the mTOR pathway was activated in tumour samples using immunohistochemical analyses of p-mTOR and p-S6k; metabolome analysis showed that the BCAAs accumulated in tumours. Recent comprehensive genetic analyses have revealed that numerous cancers harbour mutations that chronically activate the growth factor arm of mTORC1 [38]. The data presented here suggest that the high expression of BCAT1 and low expression of BCAA degradation pathways may in part be a consequence of BCAA accumulation and may promote the mTOR pathway in ICCs. Taken together, we hypothesise that upregulated BCAT1 metabolic reprogramming is a critical determinant of BCAA degradation loss, and ICC may further promote its progression by accumulating BCAAs and utilising them to activate the mTOR pathway.

Finally to determine whether the BCAA degradation pathway functionally influences the phenotype, we performed prognostic analyses of BCAT1 mRNA levels in the Shibata and our cohorts and found that the overall survival of the high BCAT1 expression group was poorer than that of the low-expression group. Moreover, we analysed the signature of the BCAA degradation pathway in the Shibata cohort. Notably, this signature negatively correlated with the 5-year survival (Fig. 6c). Thus, given a consistent rate of amino acid import, low BCAA degradation and high BCAT1 expression are key factors in ICC progression. Overall, it is possible for the set of clonal mutations to shift dramatically to ubiquitous oncogenic metabolism. The data presented here prove that the observed suppression of BCAA catabolism is not simply related to genomic alterations. These mechanisms involved in ICC progression remain a matter of debate. The sample size in our study was limited (also in that, it is not possible to investigate different etiologies). Furthermore, it will be necessary to perform further experiments with larger cohorts to validate the mechanism underlying the loss of BCAA catabolism.

In conclusion, our results provide novel insights that could lead to the development of anticancer therapeutic strategies to target cellular metabolism in cholangiocarcinoma including extrahepatic cholangiocarcinoma and gallbladder cancer, and HCC, instead of the well-known approaches for the respective molecular subtypes. Loss of BCAA catabolism with upregulated BCAT1 expression in tumours confers functional advantages, which could be exploited by therapeutic interventions to metabolic reprogramming for more ICC cases beyond genomic diversity. Therefore, the development of BCAT1 and BCAT2-specific inhibitors can be an effective antitumor strategy to improve conventional ICC therapies.

## Supplementary information

Supplementary Figure legends

Supplementary Figure and Table

Supplementary data 1

Supplementary data 2

Supplementary data 3

## Data Availability

All whole-exome and -transcriptome sequencing have been deposited in the Japanese Genotype-phenotype Archive with accession number JGAS000261.
