# Supplementary material for: Convergent genomic diversity and novel BCAA metabolism in intrahepatic cholangiocarcinoma
Source: Br J Cancer. 2023 Apr 19;128(12):2206–17. doi: 10.1038/s41416-023-02256-4 (PMC10241955; doi:10.1038/s41416-023-02256-4)
Supplement: Supplementary file 1 — Supplementary Figure legends [file 41416_2023_2256_MOESM1_ESM.docx]

**Supplementary Figure legends**

**Fig. S1. Sampling details in each case.** Intrahepatic cholangiocarcinoma (ICC) samples were obtained from 12 cases via surgical resection, resulting in a total of 77 samples, in which some of them were subjected to whole exome, RNA-seq, and proteome and metabolome analysis.

**Fig. S2A. Analysis of the number of somatic mutations.** Bar graphs show the number of ubiquitous, shared, and private mutations identified by whole exome sequencing (WES) for each sample in the 10 ICC cases.

**Fig. S2B. Mutation spectra analysis.** Mutational spectra compared between the founder and progressor mutations in the 10 ICC cases. The last two samples comprised all founder and progressor mutations. No significant differences were observed using the Wilcoxon signed-rank and Fisher’s exact tests.

**Fig. S2C. Copy number alteration (CNA) calling from our WES data.**

Heat maps showing log2-scaled ratios between normalized tumor and normal read depths (log2R) calculated from our WES data of the ten ICCs.

**Fig. S2D. Multi-region mutation profiles of ICCs except for the FS1 case.**

**Fig. S2E. Classes of cancer evolutionary trees in the simulations.** Three classes of edge lengths of the tree have been considered: trunk accumulation (green), branched accumulation (orange), and balanced accumulation (blue).

**Fig. S3A. Significant metabolic changes in multi-sampling ICC tissues except for the FS1 case.**

**Fig. S3B. Clustering of the listed tissue samples and 13 proteins**. Clustering of the tissue samples (labeled at the bottom the figure) and proteins (labeled on the right). Red and green represent increase and decrease in expression, respectively.

**Fig. S4A. Immunohistochemical detection of BCAT1 expression in a representative sample of ICC, except for the FS1 case.** T, tumor tissue; N, normal liver tissue.

**Fig. S4B. Immunohistochemical detection of BCAT2 expression in a representative sample of ICC, except for the FS1 case.** T, tumor tissue; N, normal liver tissue.

**Fig. S4C. Quantification of the immunohistochemical detection results**.

**Fig. S5.** **Summary of the BCAA degradation pathway with 36 genes in Shibata cohort (ECC, GBC, and ICC) and HCC TCGA cohort.** Clustering of tissues samples is shown towards the bottom of the figure and that of the genes is shown to the right in the figure. Red and blue coloration indicate increases and decreases in gene expression, respectively. BCAT1 and BCAT2 expression in ECC, GBC, and ICC cases and normal tissues in HCC TCGA cohort.

**Fig. S6.** **Prognostic significance of BCAT1 mRNA levels in ICC.** Kaplan-Meier survival curve of 103 ICC patients from Shibata cohort, 33 patients from TCGA cohort, and 11 ICC patients from our cohort based on BCAT1 mRNA levels.

**Fig. S7.** **Summary of BCAA catabolic enzyme CNA levels in tumor samples in our cohort.** Some genes have CNV losses and others have CNV gains. While BCAT1 shows a slight gain in CNA levels, BCAT2 shows a slight loss.

**Fig. S8.** **Heatmap of MYC module genes from ‘NEVINS_MYC_UP’ gene set.** MYC module activity was analyzed using the extraction of expression modules (EEM) algorithm in our samples. *BCAT1* mRNA levels were not explained by copy-number alteration, mRNA levels, and module activity of MYC. Color bars on the upper side of the image indicate MYC gene expression and MYC module activity. The red bar on the right side of the image indicates the MYC module genes (seed genes defined by EEM).

**Fig. S9. Correlation between MSI2 and BCTA1/BCAT2 expression in the three cohorts analyzed in this study.**

**Fig. S10. Metabolites include succinate, fumarate, and malate in 3‒4 tumor samples and one normal sample per case.**

**Fig. S11. BCAA in blood samples**

**Fig. S12. BCAA increased cell growth and invasion through mTOR in ICC cells**

**Fig. S12A**. Dose-dependent cell growth of iCC cells with BCAA.

**Fig. S12B**. Western blot analysis of the mTOR signaling pathway in BCAT1 and BCAT2 knockdown iCC (HCCC-9810) cells.

**Fig. S12C**. Cell growth of BCAT1 and BCAT2 knockdown iCC (HCCC-9810) cells. Cell growth was measured by the SRB assay in shBCAT1/shBCAT2-expressing HCCC-9810 cells.

**Fig. S13A. Immunohistochemical detection of pmTOR expression in a representative sample of ICC, except for the FS2 case.** T, tumor tissue; N, normal liver tissue.

**Fig. S13B. Immunohistochemical detection of S6K expression in a representative sample of ICC, except for the FS2 case.** T, tumor tissue; N, normal liver tissue.

**Fig. S13C Quantification of the immunohistochemical detection results**.

**Fig. S14.** **GSEA of ICC cases from GSE26566 dataset.** Enrichment plot of the correlation between BCAT1 and BCAT2 mRNA expression and HALLMARK_PI3K_AKT_MTOR_SIGNALING (NES: 1.52, p-value < 0.027 and NES: 1.22, p value= 0.18, respectively). The enrichment score (ES) reflects the degree to which a gene set is overrepresented in tumor samples. The normalized ES (NES) is calculated by normalizing the ES for each gene set to account for the size of the set.

**Fig. S15. Correlation between BCAA degradation signature and MALAT1 expression.**

**Supplementary Table S1. Detailed sampling information**

**Supplementary Table S2. BCAA degradation pathway genes**
