# Supplementary material for: Convergent genomic diversity and novel BCAA metabolism in intrahepatic cholangiocarcinoma
Source: Br J Cancer. 2023 Apr 19;128(12):2206–17. doi: 10.1038/s41416-023-02256-4 (PMC10241955; doi:10.1038/s41416-023-02256-4)
Supplement: Supplementary file 2 — Supplementary Figure and Table [file 41416_2023_2256_MOESM2_ESM.docx]

**Supplementary Materials**

**Fig. S1**


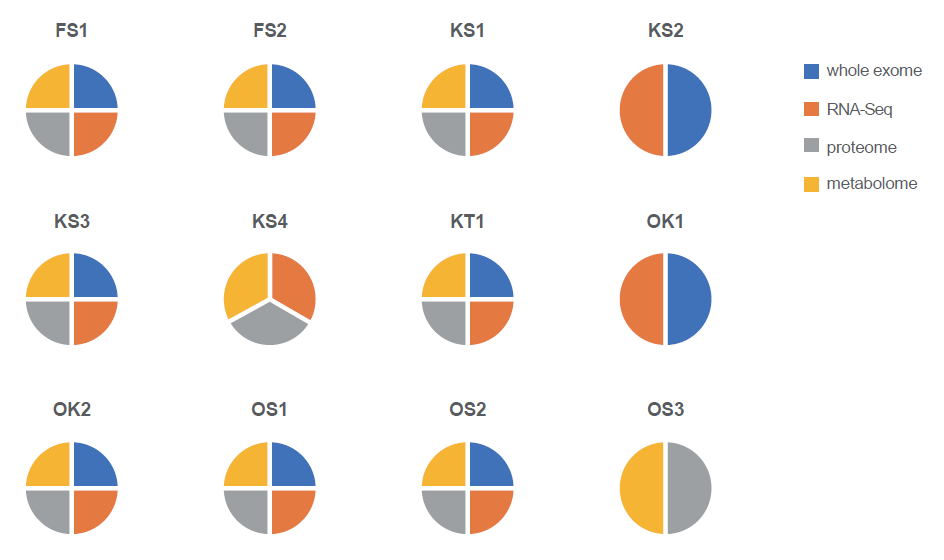


**Fig. S2A**


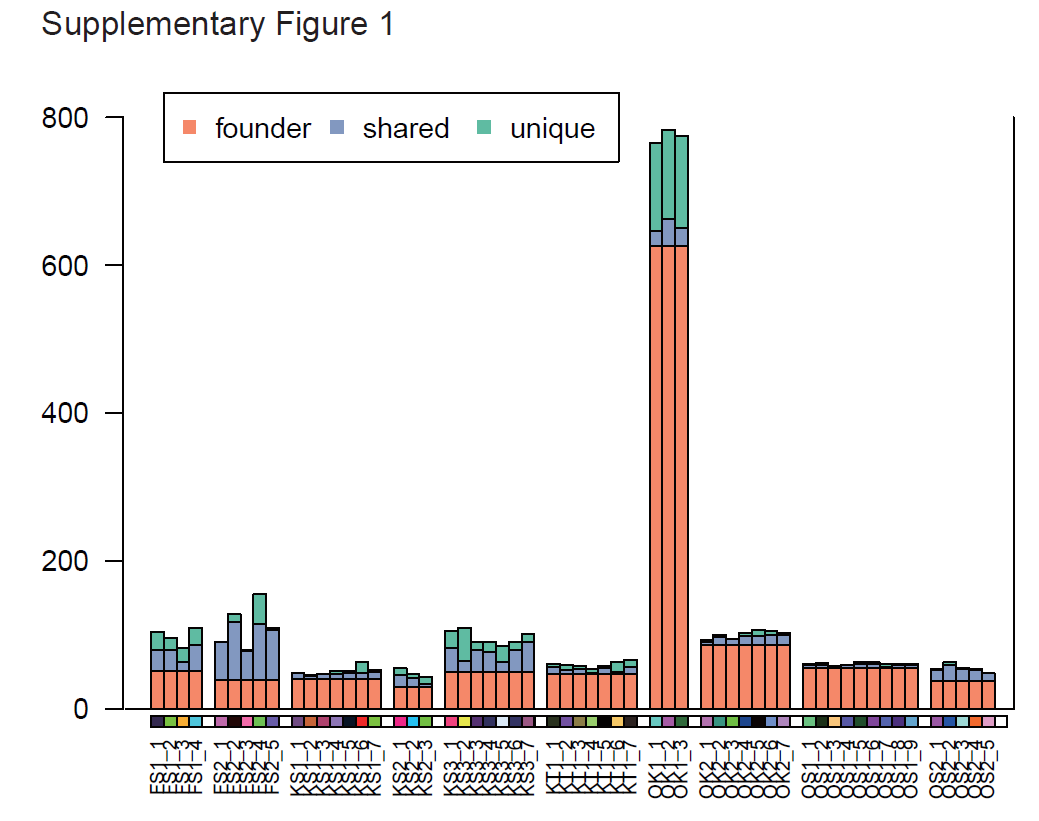


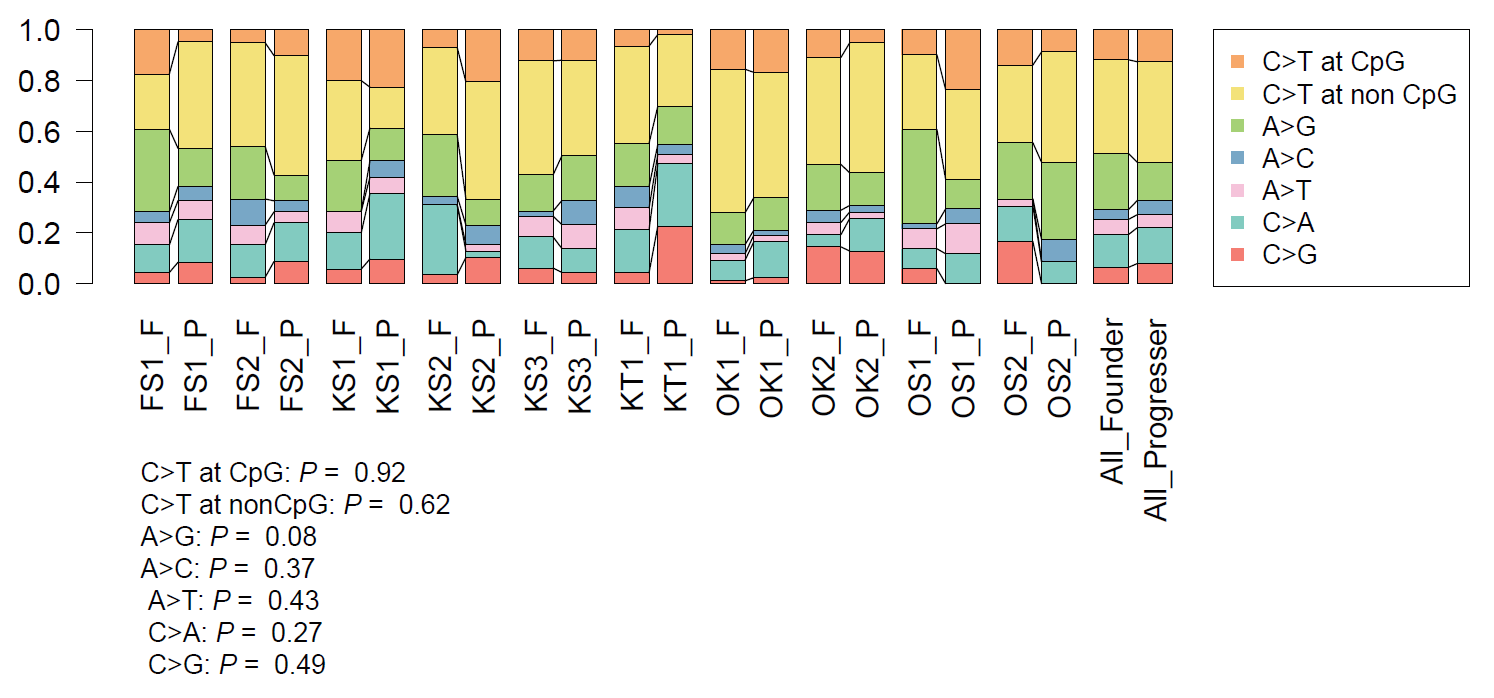
**Fig. S2B**

**Fig. S2C**


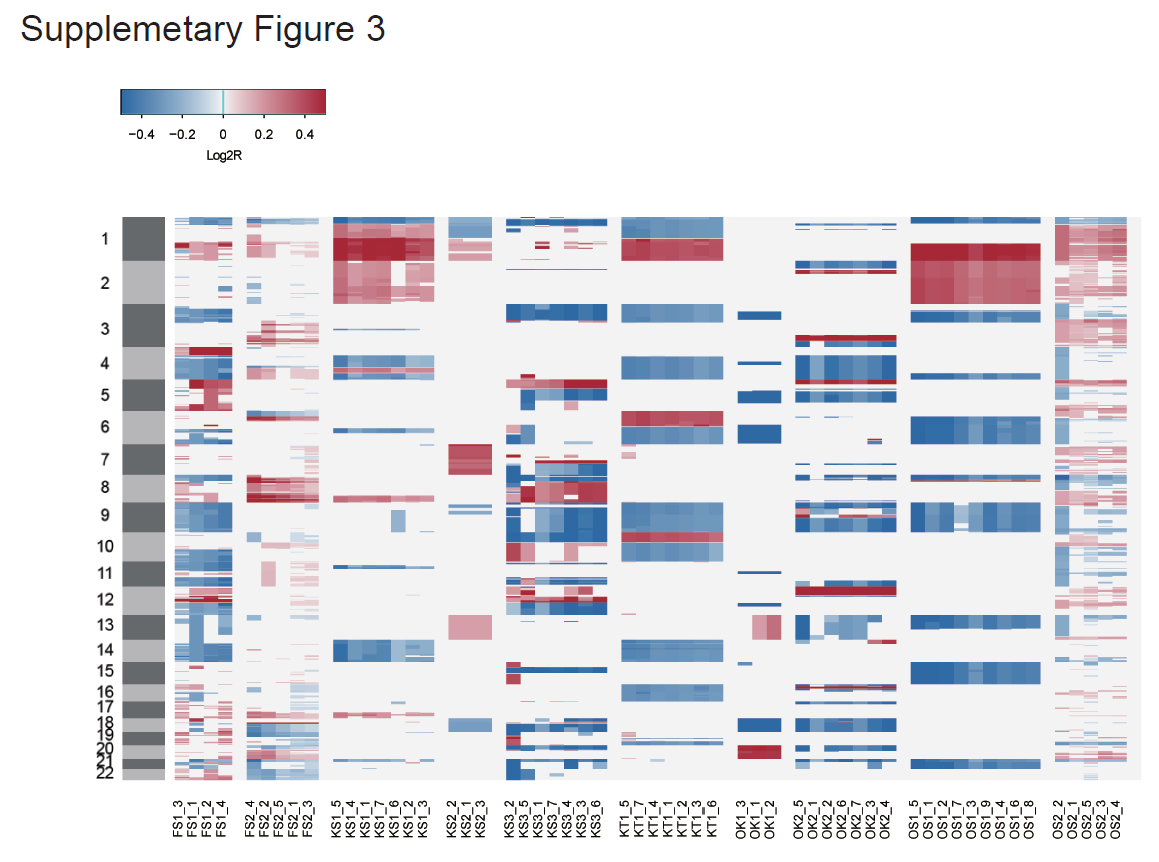


**Fig. S2D**

**
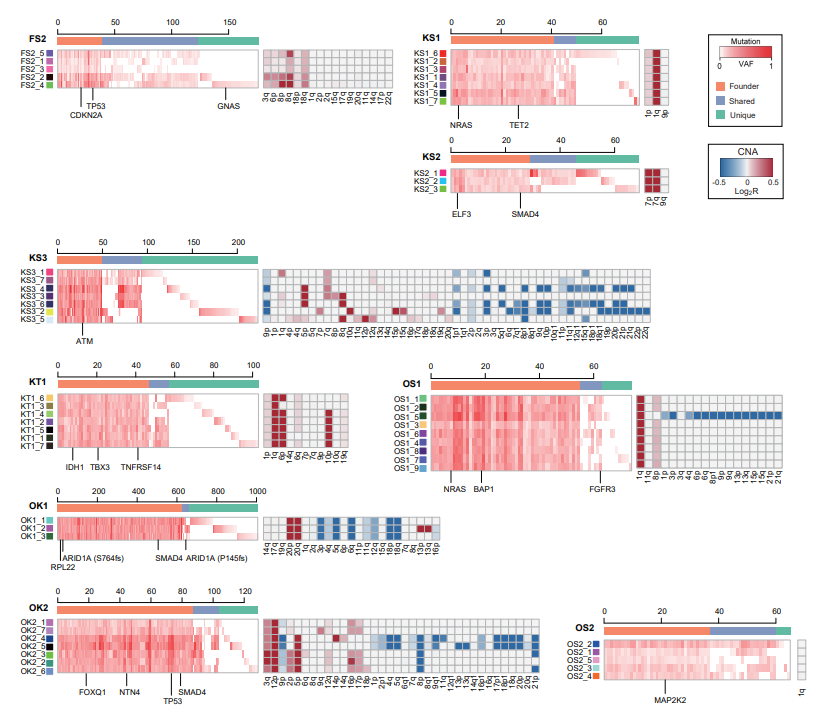
**


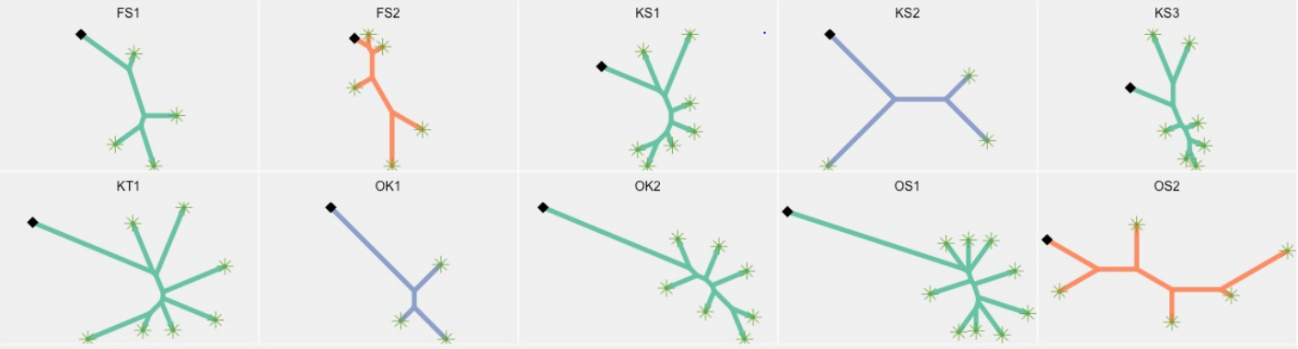
**Fig. S2E**

**Fig. S3A**

**
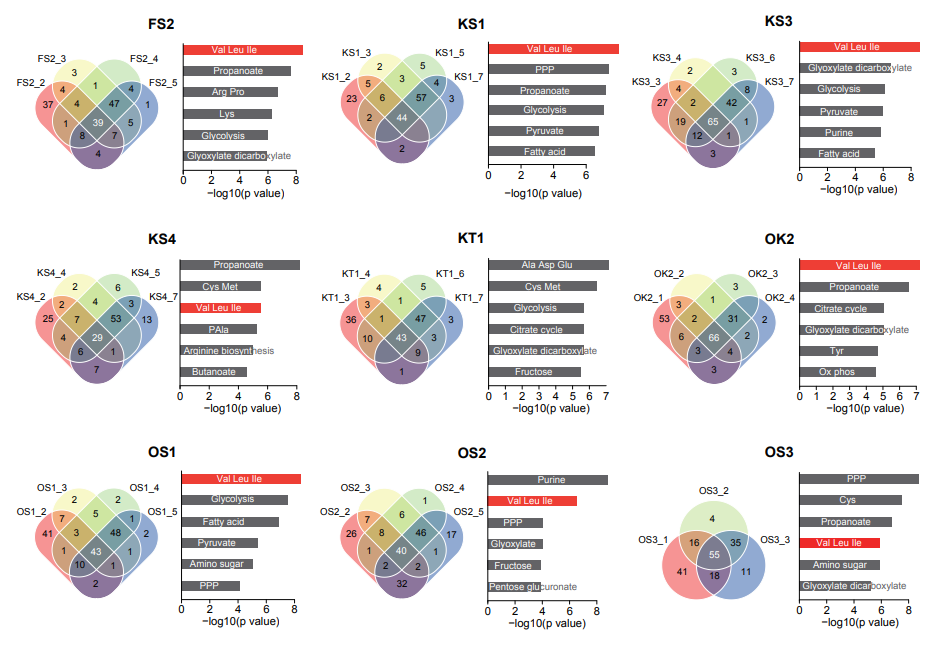
**

**Fig. S3B**
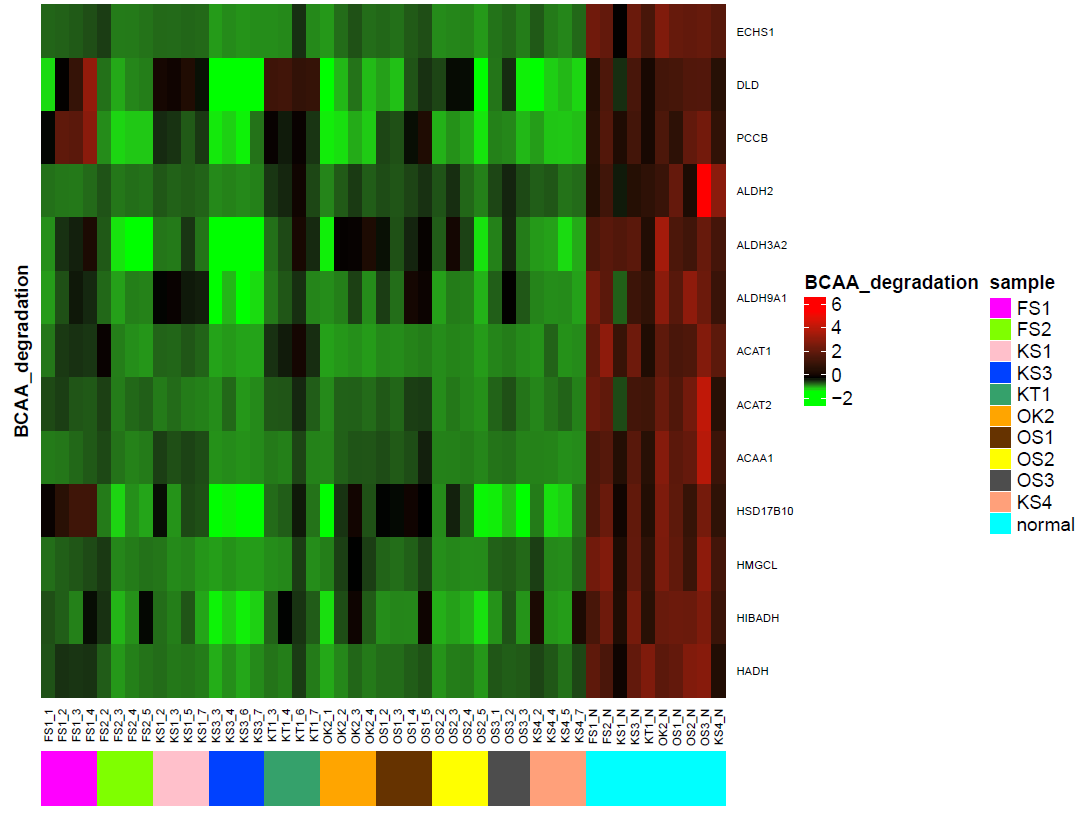


**Fig. S4A.**


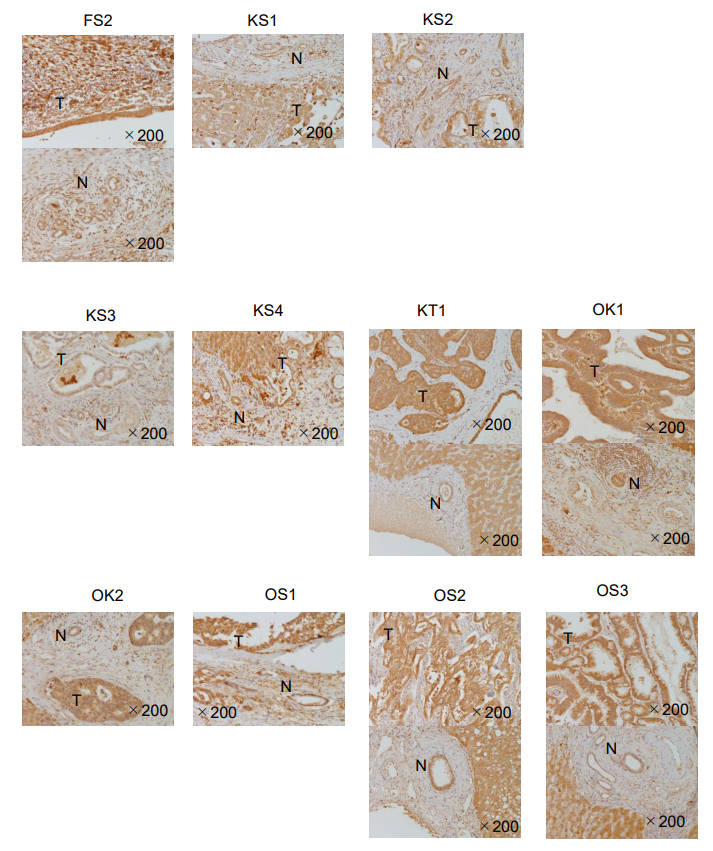


**Fig. S4B**


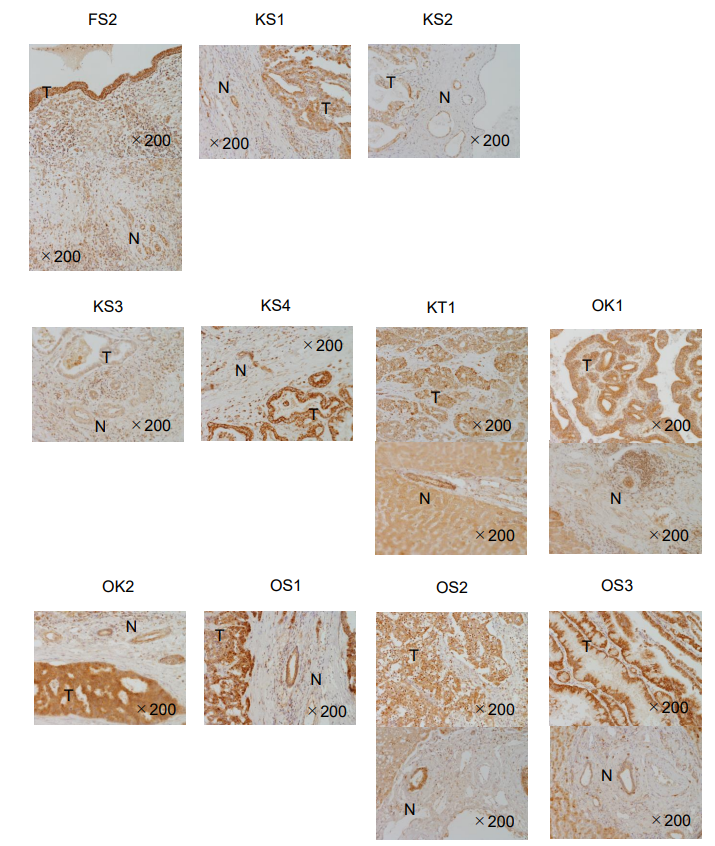


**Fig. S4C**

**Fig. S5**

**
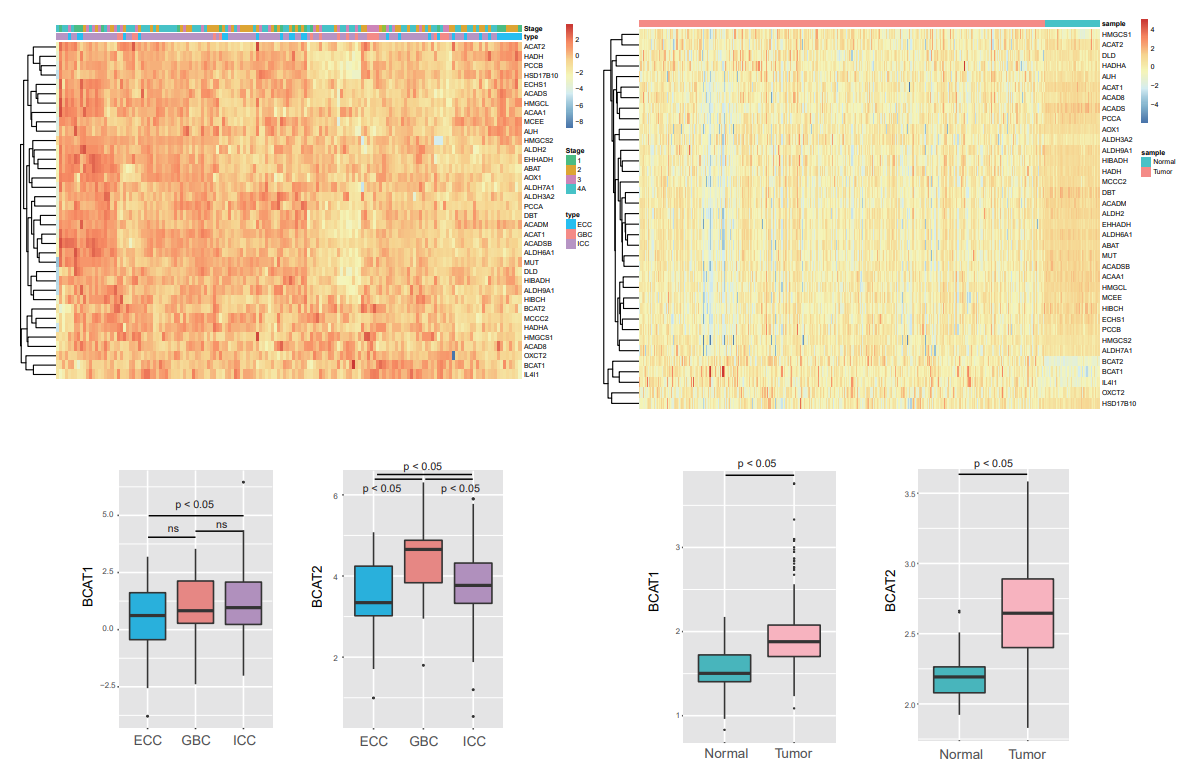
**

**Fig. S6**


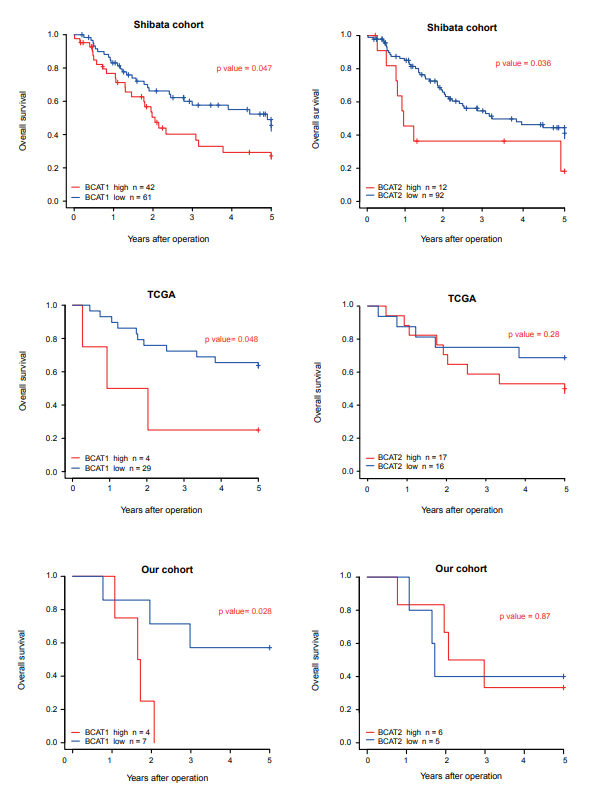


**Fig. S7**


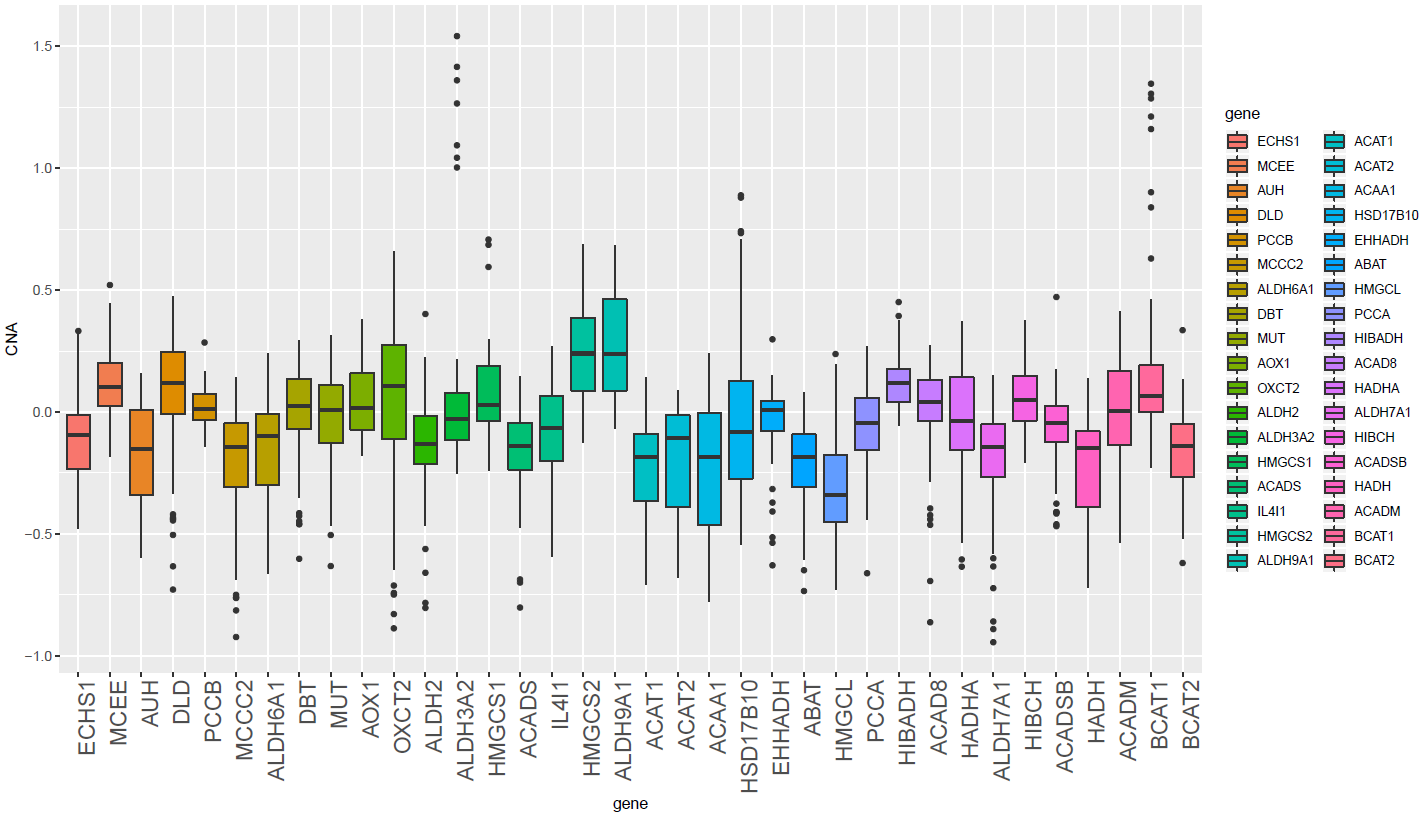


**Fig. S8**


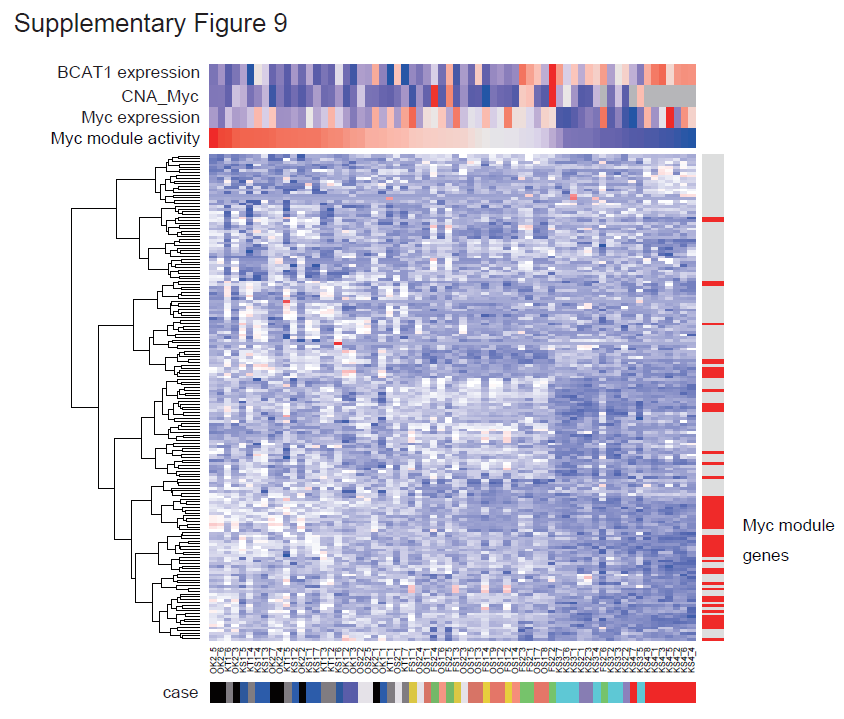


**Fig. S9**


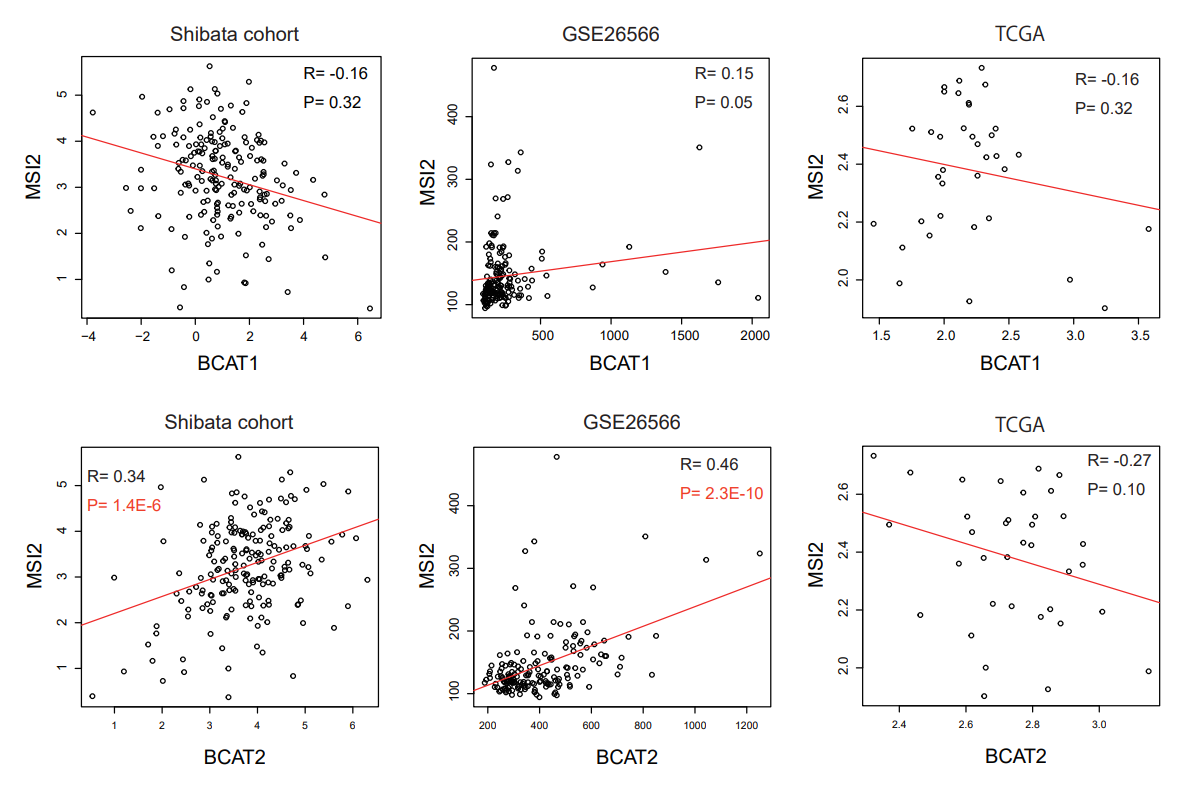


**Fig. S10**

**
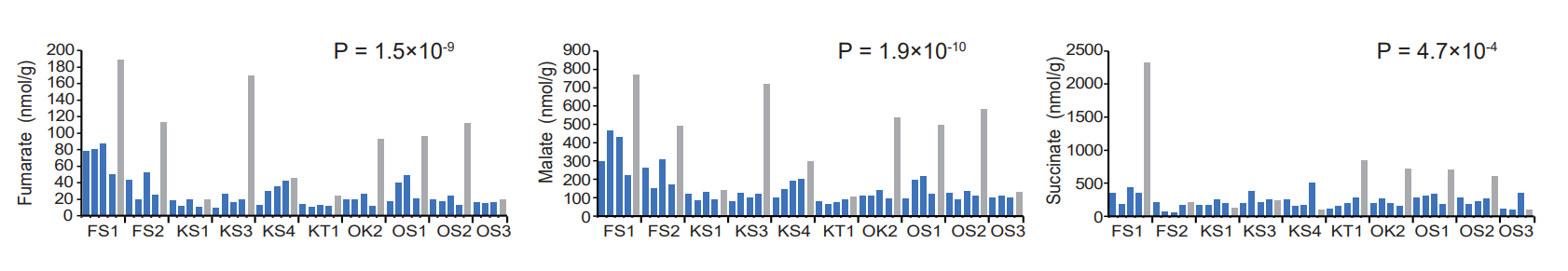
**

**Fig. S11**


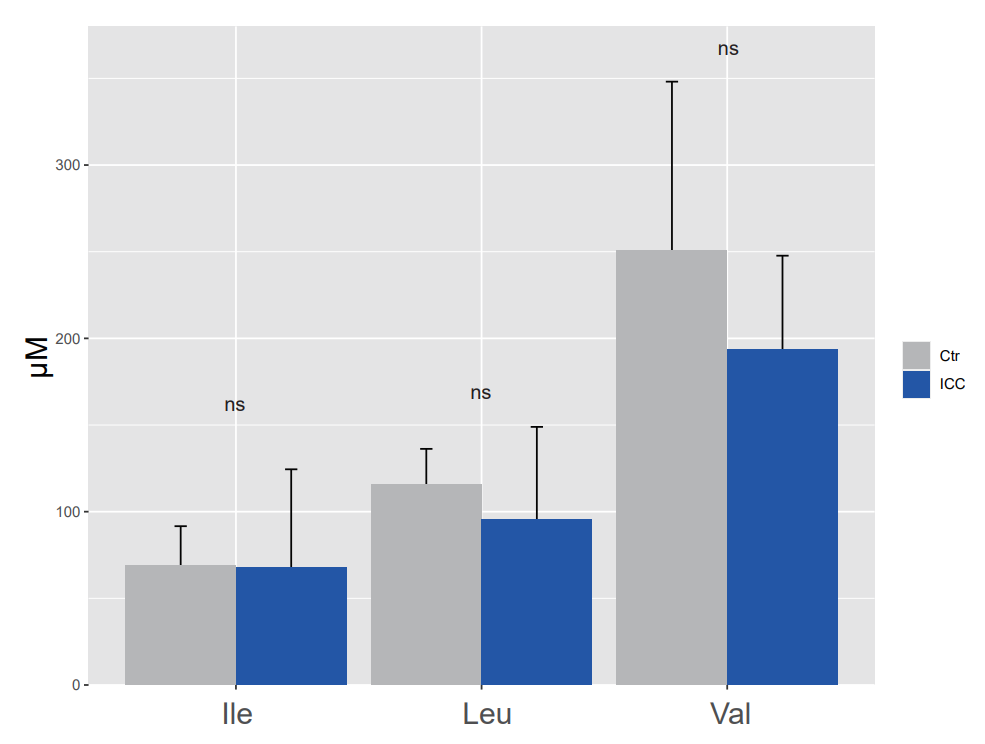


**Fig. S12**


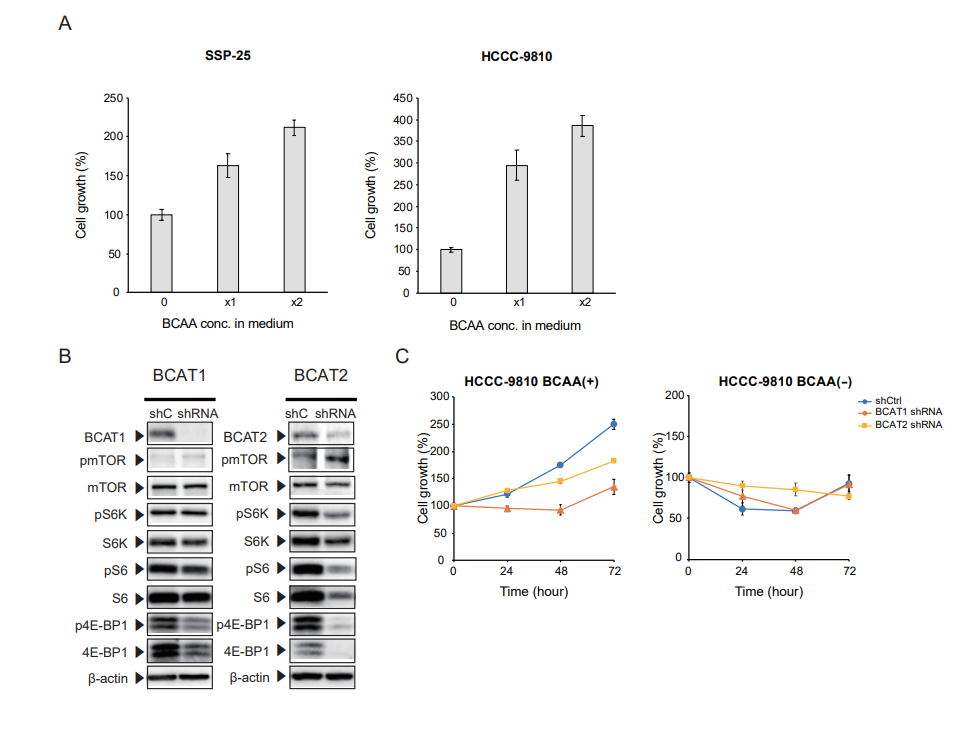


**Fig. S13A**


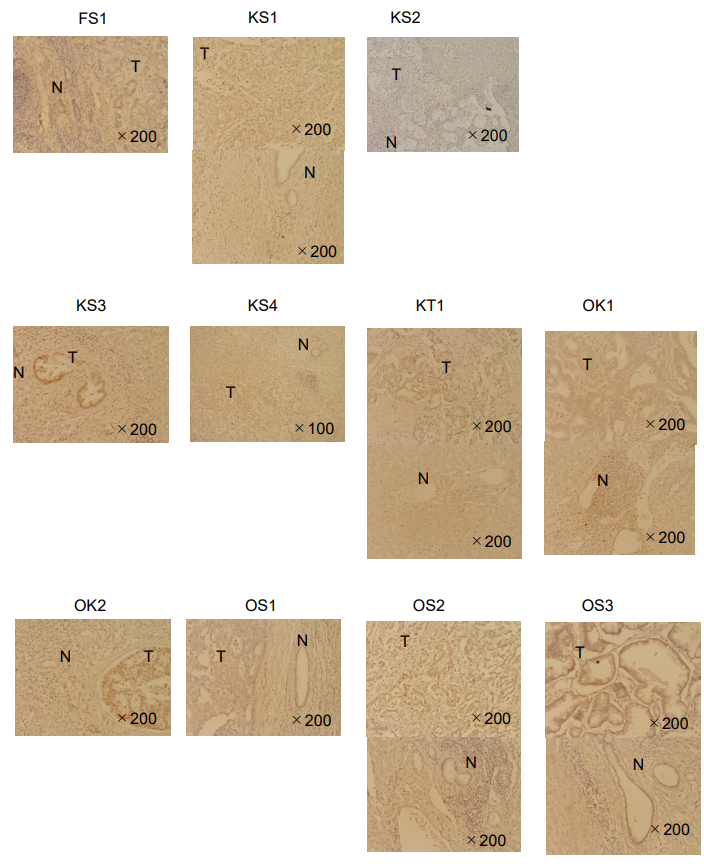


**Fig. S13B**


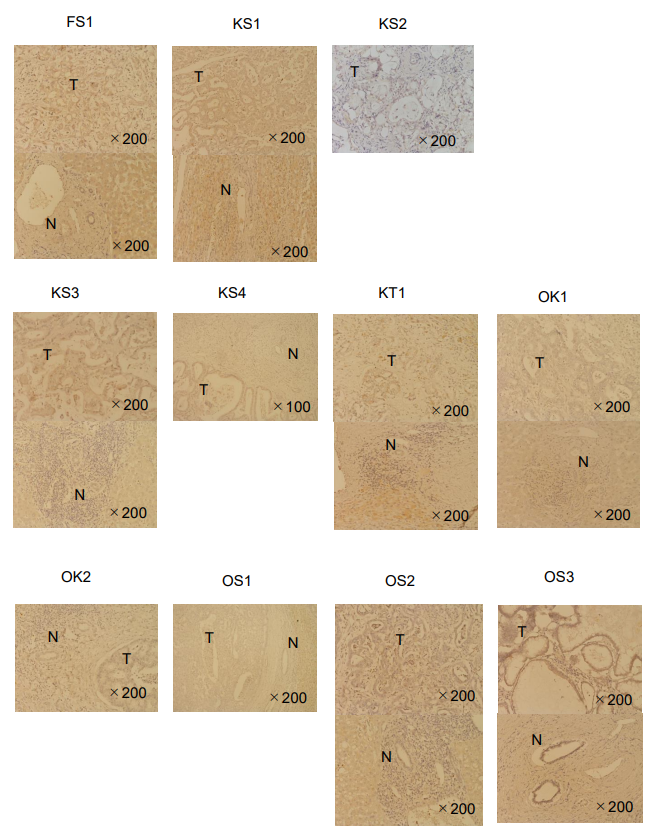


**Fig. S13C**

**Fig. S14**


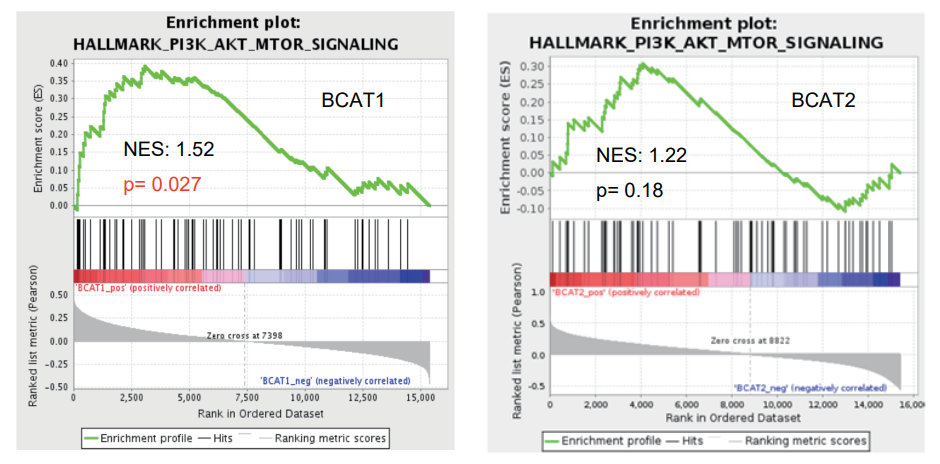


**Fig. S15**


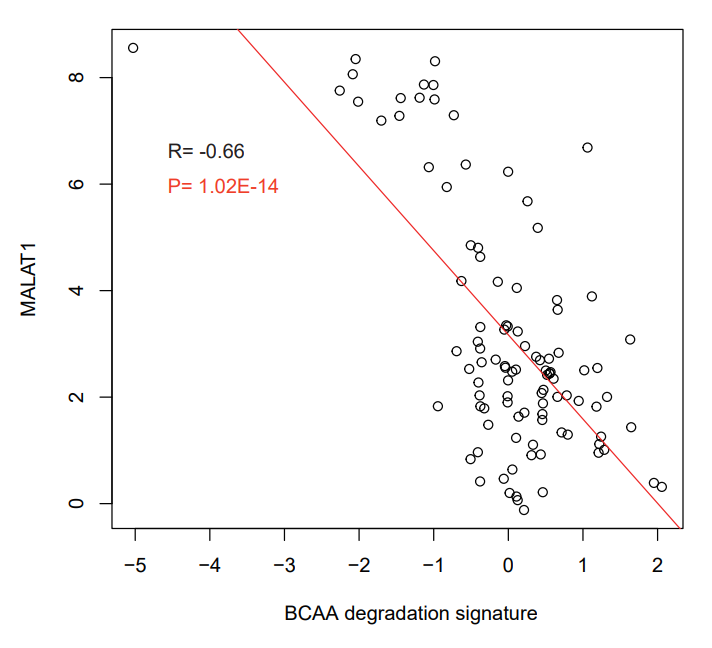


**Supplementary Table S1.**

|  | 1 | 2 | 3 | 4 | 5 | 6 | 7 | 8 | 9 | Normal |
| --- | --- | --- | --- | --- | --- | --- | --- | --- | --- | --- |
| FS1_DNA | 〇 | 〇 | 〇 | 〇 | ---- | ---- | ---- | ---- | ---- | 〇 |
| FS1_RNA | 〇 | 〇 | 〇 | 〇 | ---- | ---- | ---- | ---- | ---- | 〇 |
| FS1_Protein | 〇 | 〇 | 〇 | 〇 | ---- | ---- | ---- | ---- | ---- | 〇 |
| FS1_Metabolite | 〇 | 〇 | 〇 | 〇 | ---- |  |  | ---- | ---- | 〇 |
| FS2_DNA | 〇 | 〇 | 〇 | 〇 | 〇 | ---- | ---- | ---- | ---- | 〇 |
| FS2_RNA | 〇 | 〇 | 〇 | 〇 | 〇 | 〇 | ---- | ---- | ---- | 〇 |
| FS2_Protein | ---- | 〇 | 〇 | 〇 | 〇 | ---- | ---- | ---- | ---- | 〇 |
| FS2_Metabolite | ---- | 〇 | 〇 | 〇 | 〇 | ---- | ---- | ---- | ---- | 〇 |
| KS1_DNA | 〇 | 〇 | 〇 | 〇 | 〇 | 〇 | 〇 | ---- | ---- | 〇 |
| KS1_RNA | 〇 | 〇 | 〇 | 〇 | 〇 | 〇 | 〇 | ---- | ---- | 〇 |
| KS1_Protein | ---- | 〇 | 〇 | ---- | 〇 | ---- | 〇 | ---- | ---- | 〇 |
| KS1_Metabolite | ---- | 〇 | 〇 | ---- | 〇 | ---- | 〇 | ---- | ---- | 〇 |
| KS2_DNA | 〇 | 〇 | 〇 | ---- | ---- | ---- | ---- | ---- | ---- | 〇 |
| KS2_RNA | 〇 | 〇 | 〇 | ---- | ---- | ---- | ---- | ---- | ---- | 〇 |
| KS2_Protein | ---- | ---- | ---- | ---- | ---- | ---- | ---- | ---- | ---- | 〇 |
| KS2_Metabolite | ---- | ---- | ---- | ---- | ---- | ---- | ---- | ---- | ---- | ---- |
| KS3_DNA | 〇 | 〇 | 〇 | 〇 | 〇 | 〇 | 〇 | ---- | ---- | 〇 |
| KS3_RNA | 〇 | 〇 | 〇 | 〇 | 〇 | 〇 | 〇 | ---- | ---- | 〇 |
| KS3_Protein | ---- | ---- | 〇 | 〇 | ---- | 〇 | 〇 | ---- | ---- | 〇 |
| KS3_Metabolite | ---- | ---- | 〇 | 〇 | ---- | 〇 | 〇 | ---- | ---- | 〇 |
| KS4_DNA | ---- | ---- | ---- | ---- | ---- | ---- | ---- | ---- | ---- | 〇 |
| KS4_RNA | 〇 | 〇 | 〇 | 〇 | 〇 | 〇 | 〇 | 〇 | ---- | 〇 |
| KS4_Protein | ---- | 〇 | ---- | 〇 | 〇 | ---- | 〇 | ---- | ---- | 〇 |
| KS4_Metabolite | ---- | 〇 | ---- | 〇 | 〇 | ---- | 〇 | ---- | ---- | 〇 |
| KT1_DNA | 〇 | 〇 | 〇 | 〇 | 〇 | 〇 | 〇 | ---- | ---- | 〇 |
| KT1_RNA | 〇 | 〇 | 〇 | 〇 | 〇 | 〇 | 〇 | ---- | ---- | 〇 |
| KT1_Protein | ---- | ---- | 〇 | 〇 | ---- | 〇 | 〇 | ---- | ---- | 〇 |
| KT1_Metabolite | ---- | ---- | 〇 | 〇 | ---- | 〇 | 〇 | ---- | ---- | 〇 |
| OK1_DNA | 〇 | 〇 | 〇 | ---- | ---- | ---- | ---- | ---- | ---- | 〇 |
| OK1_RNA | 〇 | 〇 | 〇 | ---- | ---- | ---- | ---- | ---- | ---- | 〇 |
| OK1_Protein | ---- | ---- | ---- | ---- | ---- | ---- | ---- | ---- | ---- | ---- |
| OK1_Metabolite | ---- | ---- | ---- | ---- | ---- | ---- | ---- | ---- | ---- | ---- |
| OK2_DNA | 〇 | 〇 | 〇 | 〇 | 〇 | 〇 | 〇 | ---- | ---- | 〇 |
| OK2_RNA | 〇 | 〇 | 〇 | 〇 | 〇 | 〇 | 〇 | ---- | ---- | 〇 |
| OK2_Protein | 〇 | 〇 | 〇 | 〇 | ---- | ---- | ---- | ---- | ---- | 〇 |
| OK2_Metabolite | 〇 | 〇 | 〇 | 〇 | ---- | ---- | ---- | ---- | ---- | 〇 |
| OS1_DNA | 〇 | 〇 | 〇 | 〇 | 〇 | 〇 | 〇 | 〇 | 〇 | 〇 |
| OS1_RNA | 〇 | 〇 | 〇 | 〇 | 〇 | 〇 | 〇 | 〇 | 〇 | 〇 |
| OS1_Protein | ---- | 〇 | 〇 | 〇 | 〇 | ---- | ---- | ---- | ---- | 〇 |
| OS1_Metabolite | ---- | 〇 | 〇 | 〇 | 〇 | ---- | ---- | ---- | ---- | 〇 |
| OS2_DNA | 〇 | 〇 | 〇 | 〇 | 〇 | ---- | ---- | ---- | ---- | 〇 |
| OS2_RNA | 〇 | 〇 | 〇 | 〇 | 〇 | ---- | ---- | ---- | ---- | 〇 |
| OS2_Protein | ---- | 〇 | 〇 | 〇 | 〇 | ---- | ---- | ---- | ---- | 〇 |
| OS2_Metabolite | ---- | 〇 | 〇 | 〇 | 〇 | ---- | ---- | ---- | ---- | 〇 |
| OS3_DNA | ---- | ---- | ---- | ---- | ---- | ---- | ---- | ---- | ---- | ---- |
| OS3_RNA | ---- | ---- | ---- | ---- | ---- | ---- | ---- | ---- | ---- | ---- |
| OS3_Protein | 〇 | 〇 | 〇 | ---- | ---- | ---- | ---- | ---- | ---- | 〇 |
| OS3_Metabolite | 〇 | 〇 | 〇 | ---- | ---- | ---- | ---- | ---- | ---- | 〇 |

**Supplementary Table S2.**

AOX1

ALDH1B1

ACADS

ACADSB

ABAT

ALDH2

ACADM

ACAT2

HSD17B10

ACAT1

OXCT1

ACAA2

IL4I1

HADH

MCEE

HADHB

DBT

HADHA

ALDH6A1

HMGCL

IVD

BCKDHB

ACAD8

ECHS1

ALDH9A1

ALDH3A2

PCCB

HIBCH

DLD

HMGCS2

EHHADH

HMGCS1

PCCA

ALDH7A1

HIBADH

ACAA1

OXCT2

MUT

MCCC2

BCAT1

AUH

BCAT2

BCKDHA

MCCC1
